# Experimental validation of methods for differential gene expression analysis and sample pooling in RNA-seq

**DOI:** 10.1186/s12864-015-1767-y

**Published:** 2015-07-25

**Authors:** Anto P. Rajkumar, Per Qvist, Ross Lazarus, Francesco Lescai, Jia Ju, Mette Nyegaard, Ole Mors, Anders D. Børglum, Qibin Li, Jane H. Christensen

**Affiliations:** Department of Biomedicine, Aarhus University, 6, Bartholins Allé, Aarhus C, Aarhus, 8000 Denmark; Mental Health of Older Adults and Dementia Clinical Academic Group, South London and Maudsley NHS foundation trust, London, UK; Wolfson Centre for Age Related Diseases, Institute of Psychiatry, Psychology, & Neuroscience, King’s College, London, UK; The Initiative for Integrative Psychiatric Research, iPSYCH, Aarhus, 8000 Denmark; Center for Integrative Sequencing, iSEQ, Aarhus University, Aarhus, 8000 Denmark; Computational Biology, Baker IDI heart and diabetes institute, Victoria, 8008 Australia; Beijing Genomics Institute, Shenzhen, 518083 China; Research Department P, Aarhus University Hospital, Risskov, Denmark; Translational Neuropsychiatry Unit, Aarhus University, Aarhus, 8240 Denmark

**Keywords:** Gene expression, Next-generation RNA Sequencing, Predictive value of tests, Quantitative real-time polymerase chain reaction, Sensitivity and specificity

## Abstract

**Background:**

Massively parallel cDNA sequencing (RNA-seq) experiments are gradually superseding microarrays in quantitative gene expression profiling. However, many biologists are uncertain about the choice of differentially expressed gene (DEG) analysis methods and the validity of cost-saving sample pooling strategies for their RNA-seq experiments. Hence, we performed experimental validation of DEGs identified by Cuffdiff2, edgeR, DESeq2 and Two-stage Poisson Model (TSPM) in a RNA-seq experiment involving mice amygdalae micro-punches, using high-throughput qPCR on independent biological replicate samples. Moreover, we sequenced RNA-pools and compared their results with sequencing corresponding individual RNA samples.

**Results:**

False-positivity rate of Cuffdiff2 and false-negativity rates of DESeq2 and TSPM were high. Among the four investigated DEG analysis methods, sensitivity and specificity of edgeR was relatively high. We documented the pooling bias and that the DEGs identified in pooled samples suffered low positive predictive values.

**Conclusions:**

Our results highlighted the need for combined use of more sensitive DEG analysis methods and high-throughput validation of identified DEGs in future RNA-seq experiments. They indicated limited utility of sample pooling strategies for RNA-seq in similar setups and supported increasing the number of biological replicate samples.

**Electronic supplementary material:**

The online version of this article (doi:10.1186/s12864-015-1767-y) contains supplementary material, which is available to authorized users.

## Background

Massively parallel cDNA sequencing (RNA-seq) is gradually superseding microarrays in quantitative gene expression profiling [1]. Apart from its ability to detect novel transcripts, splicing events, and sequence variations, RNA-seq offers unparalleled precise detection of gene expression over a wide dynamic range [2]. Due to declining costs of sequencing, further increase in the use of RNA-seq is expected. However, several methodological [3] and statistical [4] issues in the design and analyses of RNA-seq experiments remain unresolved. Biologists, who plan RNA-seq experiments, often pose questions on sample size requirements, cost-effective strategies for sample pooling, and on the choice of data analysis software. Current literature on this topic [4–10] do not provide unequivocal answers to these important questions [1].

Diverse methods are available to align RNA-seq reads [7], summarize read counts, assemble transcripts [8], and to detect differential expression between biological conditions [11]. Differentially expressed gene (DEG) analysis methods differ on their normalisation procedures, detection of differential isoform expression, statistical modelling, variance estimation, and corrections for multiple testing [4]. This research focussed on four commonly used DEG analysis methods, Cuffdiff2 [12], edgeR [13], DESeq2 [14], and Two-stage Poisson Model (TSPM) [15]. It minimised the variations, secondary to read alignment and counting, by employing the same spliced aligner (Tophat2) [16] and counting algorithm (HTSeq 0.5.4) [17] for all methods, except for Cuffdiff2 that requires a unique quantification method, Cufflinks [18]. We analysed data, derived from a RNA-seq experiment including RNA samples, extracted from amygdalae micro-punches of a genetically modified mouse strain (*Brd1*^+/−^) and of their wild-type (WT) littermates (8 biological replicates/group), with Cuffdiff2, edgeR, DESeq2 and TSPM. Validation using independent biological replicates is preferred over *in silico* analyses, using online databases or simulated datasets, as well as technical validation, using the same RNA samples, to confirm true-positive DEGs between two or more biological conditions [19, 20]. Hence, we validated the differential expression of 115 genes, randomly selected from the list of DEGs that were identified by the four methods, using independent biological replicates and high-throughput quantitative reverse-transcription PCR (qPCR).

Pooling biological replicate RNA samples, such as those derived from a number of experimentally similar animals, may retain the biological information, while reducing the cost of sequencing. Validity and utility of sample pooling for gene expression analyses using microarrays have been evaluated extensively [21–23]. Biological averaging hypothesis suggests reduced biological variability and increased power to detect DEGs [21], but a pooling bias, that is the difference between the value measured in the pool and the mean of the values measured in the corresponding individual replicates, can occur [24]. Although several RNA-seq experiments, based on pooled samples of RNA, have identified DEGs [25], the validity of pooling for RNA-seq experiments to detect DEGs has not been systematically evaluated so far. Hence, we evaluated the validity of two pooling strategies (3 or 8 biological replicates/pool; two pools/group) against the reference standard of sequencing the corresponding individual samples (3 or 8 samples/group) to detect DEGs.

## Results

### Validity of DEG analysis methods

**Fig. 1:**
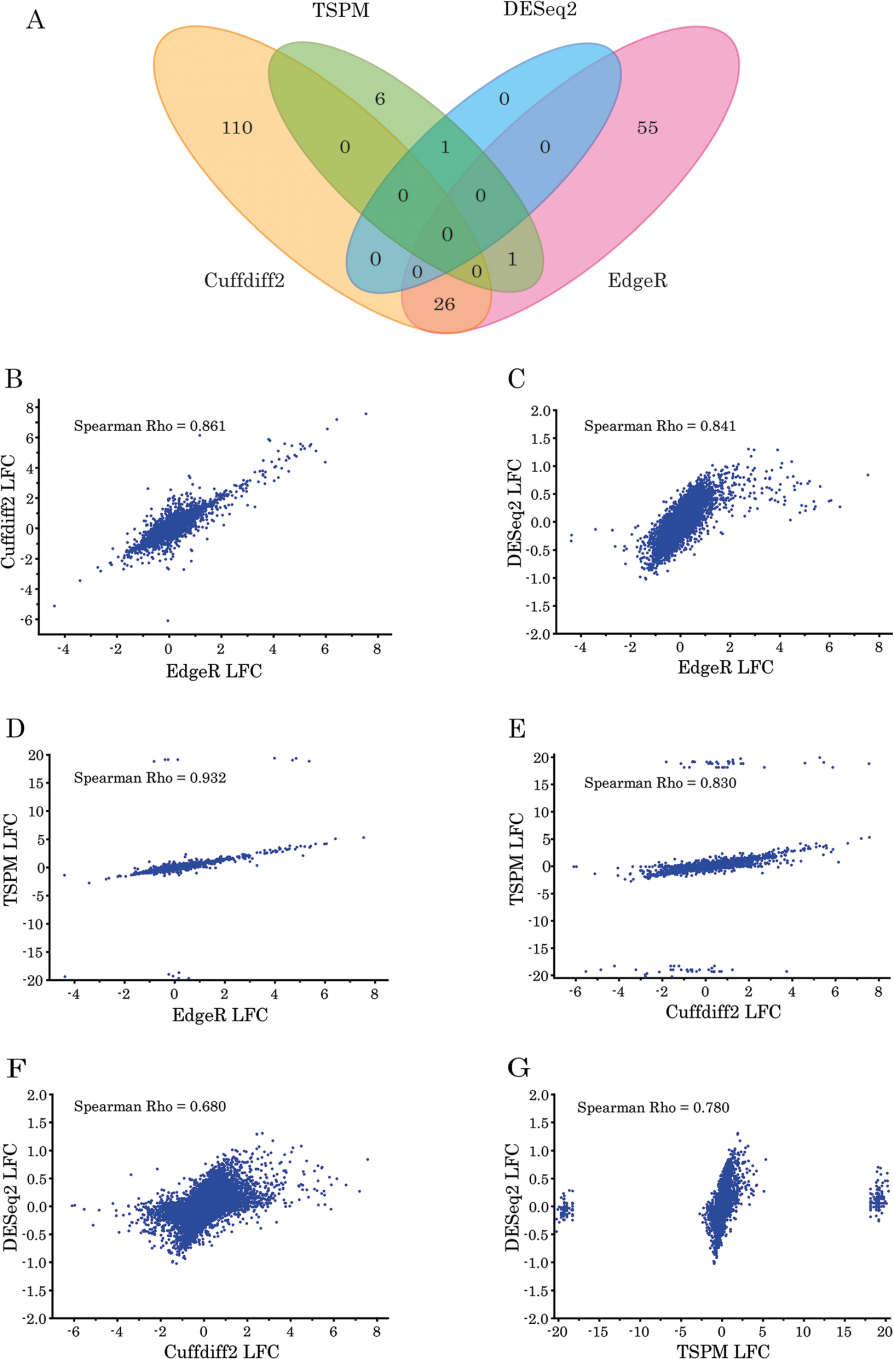
Agreement between four different methods for DEG analysis of RNA-seq data. **a** Intersections between DEGs, which were detected by Cuffdiff2, edgeR, DESeq2 and Two-stage Poisson Model (TSPM), after Benjamini-Hochberg false discovery correction at 5 %. **b-g** Pairwise comparisons of logarithmic (base 2) fold changes (LFC) in expression that were estimated by Cuffdiff2, edgeR, DESeq2 and TSPM: **b** edgeR and Cuffdiff2; **c** edgeR and DESeq2; **d** edgeR and TSPM; **e** Cuffdiff2 and TSPM; **f** Cuffdiff2 and DESeq2; **g** TSPM and DESeq2; Spearman correlation coefficients (Rho) are included in each graph. RNA samples were obtained from amygdalae micro-punches of female mice, heterozygous for a targeted deletion in the *Brd1* gene on a congenic C57BL/6NTac background and of their WT littermates (8 biological replicates/group)

We performed differential gene expression analysis of data from a RNA-seq experiment using Cuffdiff2, edgeR, DESeq2 and TSPM. After Benjamini-Hochberg false discovery correction, genes with adjusted p values less than 0.05 were considered as DEGs for all methods. Figure 1 presents the agreement between the four analysis methods. 199 DEGs were identified in total. Cuffdiff2 detected more DEGs than the other methods, while DESeq2 identified only *Brd1* as a DEG. None of the DEGs was identified by any three of these methods (Fig. 1a). Spearman correlation coefficients between the logarithmic (base 2) fold changes in expression (LFC), estimated by these methods, ranged from 0.680 to 0.932 (p < 0.0001 for each correlation) (Fig. 1b-g). However, the range of estimated fold changes varied, especially for the genes that were expressed in only one group (Fig. 1b-g). Cuffdiff2 assigned infinite values for the LFC of the genes, expressed in only one group, while the other three methods estimated values ranging from 0 to ±20 for them. The digital expression count matrix [Additional file 1] and complete Cuffdiff2 [Additional file 2], DESeq2 [Additional file 3] and TSPM [Additional file 4] analyses are included as additional data files with the online version of this paper. edgeR results will be published elsewhere. Among 115 randomly selected genes from the list of 199 DEGs, detected by Cuffdiff2, edgeR, DESeq2 and TSPM, 60 were replicated in biological independent RNA samples by qPCR, while other 55 failed replication [Additional file 5]. Table 1 presents the sensitivity, specificity, predictive values and likelihood ratios of Cuffdiff2, edgeR, DESeq2 and TSPM, assuming qPCR as the reference standard. DESeq2 was the most specific (100 %), but the least sensitive method (1.67 %). Cuffdiff2 identified more than half (51.67 %) of the true-positive DEGs, but contributed 87 % of the false positive DEGs. edgeR displayed the best sensitivity (76.67 %) and overall agreement with a false positivity rate of 9 %. TSPM had specificity, comparable to edgeR (90.91 %), but showed a false negativity rate of 95 %. Positive predictive values of Cuffdiff2, DESeq2, edgeR, and TSPM were 39.24 %, 100 %, 90.20 %, 37.50 %, respectively. Combining edgeR and Cuffdiff2 analyses in parallel enhanced sensitivity to 88.72 %, but their net specificity was only 11.57 %. Spearman correlation coefficients between the LFCs, estimated by qPCR, and those estimated by edgeR, Cuffdiff2, DESeq2 and TSPM were 0.541, 0.524, 0.453 and 0.511, respectively (p < 0.001) [Additional file 6]. Root-mean-square deviation accuracies of edgeR, Cuffdiff2, DESeq2 and TSPM with reference to qPCR LFC were 1.88, 2.11, 1.18 and 2.50, respectively.

**Table 1:** Validation of four differential gene expression analysis methods for RNA-Seq

Parameters^a^	edgeR	Cuffdiff2	TSPM	DESeq2
Total number of identified DEGs^b^	82	136	8	1
Number for DEGs selected for qPCR validation	51	79	8	1
Sensitivity (True positivity rate) (%)	76.67	51.67	5.00	1.67
Specificity (True negativity rate) (%)	90.91	12.73	90.91	100.00
False positivity rate (%)	9.09	87.27	9.09	0.00
False negativity rate (%)	23.33	48.33	95.00	98.33
Positive predictive value (%)	90.20	39.24	37.50	100.00
Negative predictive value (%)	78.13	19.44	46.73	48.25
Positive likelihood ratio	8.43	0.59	0.55	∞
Negative Likelihood ratio	0.26	3.80	1.05	0.98
Overall agreement (%)	83.48	33.04	46.09	48.70

^a^Replication of differential expression by quantitative Polymerase Chain Reaction (qPCR) was the reference standard

^b^Differentially Expressed Genes, after Benjamini-Hochberg false discovery correction at 5 %; TSPM: Two-stage Poisson Model

### Validity of RNA pooling for DEG analyses

**Fig. 2:**
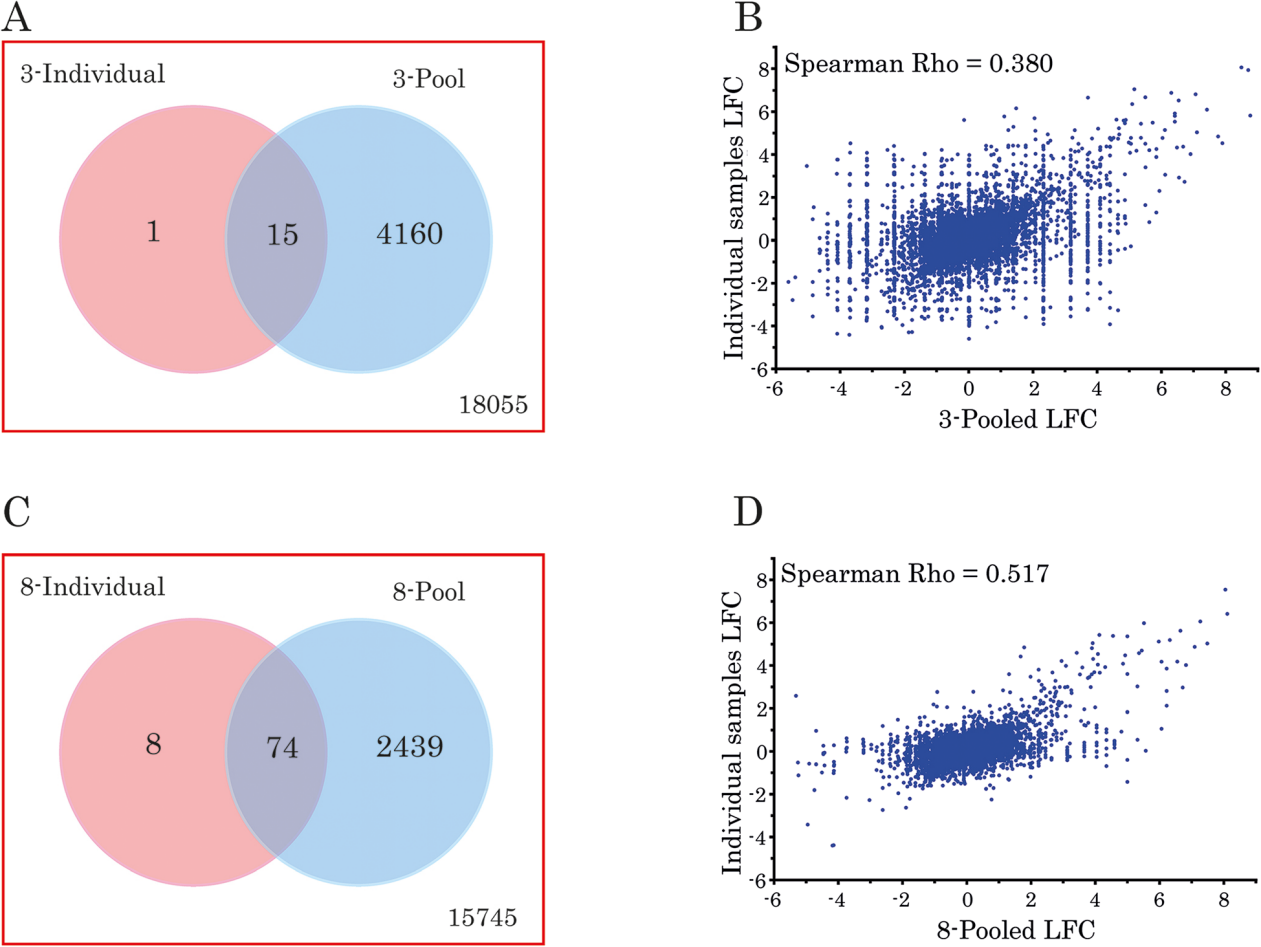
Agreement between sequencing RNA-pools and sequencing corresponding individual RNA samples. **a** Intersection between differentially expressed genes (DEGs), detected by edgeR, in RNA-seq data from pooled RNA (3 samples/ pool; two pools/ group) and of data from corresponding individual samples of RNA (3 samples/group). Rectangle represents all expressed genes. **b** Correlation between the logarithmic (base 2) fold changes (LFC) in expression that were estimated by sequencing RNA-pools (3 samples/ pool) and by sequencing corresponding individual samples (3 samples/group). **c** Intersection between the DEGs, detected by edgeR, in RNA-seq data from pooled RNA (8 samples/ pool; two pools/ group) and of data from corresponding individual samples of RNA (8 samples/group). Rectangle represents all expressed genes. **d** Correlation between the LFC in expression that were estimated by sequencing RNA-pools (8 samples/pool) and by sequencing corresponding individual samples (8 samples/ group)

RNA-seq data from RNA-pools were analysed by edgeR [Additional file 7] and the results were compared with edgeR analyses of RNA-seq data from the corresponding individual RNA samples. Figure 2 presents this comparison. Analyses detected 4175 and 2513 DEGs in 3-sample and 8-sample pools of RNA, respectively. Differential expression of most of these genes was not corroborated by the analyses of corresponding individual samples. Agreement between the analyses of RNA-pools and of corresponding individual samples was weak (Cohen’s κ < 0.05). Table 2 presents the sensitivity, specificity and predictive values of the two pooling strategies, assuming the edgeR analyses of the corresponding individual samples as the reference standard. Despite having good sensitivity (93.75 % and 90.24 %, respectively) and specificity (81.27 % and 86.59 %, respectively), both pooling strategies displayed poor positive predictive values (0.36 % and 2.94 %, respectively), which undermined their ability to predict true-positive DEGs. Adding eight (Spearman ρ = 0.517; p < 0.0001), instead of three (Spearman ρ = 0.380; p < 0.0001), biological replicates to the RNA-pool significantly improved the correlation between the LFC, estimated by the analyses of pooled and the corresponding individual samples (Z = 17.25; p < 0.0001). We repeated similar analyses with Cuffdiff2 [Additional file 8], and replicated poor positive predictive values of both pooling strategies [Additional file 9].

**Table 2:** Validation of two pooling strategies for RNA-Seq

Parameters^a^	Pooling 3 samples	Pooling 8 samples
Total number of identified DEGs^b^	4175	2513
Sensitivity (True positivity rate) (%)	93.75	90.24
Specificity (True negativity rate) (%)	81.27	86.59
False positivity rate (%)	18.73	13.41
False negativity rate (%)	6.25	9.76
Positive predictive value (%)	0.36	2.94
Negative predictive value (%)	99.99	99.95
Agreement between identified DEGs^c^	0.006	0.049
Correlation between reported LFC^d^	0.380	0.517
Root-mean-square deviation of LFC^e^	1.198	0.518

^a^Sequencing corresponding individual biological samples was the reference standard

^b^Differentially Expressed Genes (DEGs), after Benjamini-Hochberg false discovery correction at an expected rate of 5 %

^c^Inter-rater agreement Cohen’s kappa between sequencing individual samples (3 or 8/group) and sequencing pooled samples (3 or 8 biological replicates/pool; 2 pools/group) to identify DEGs

^d^Spearman correlation coefficient between the logarithmic fold changes (LFC), which were estimated by sequencing individual samples and by sequencing pooled samples

^e^Standard deviation of the differences between the LFC, estimated by sequencing individual samples and by sequencing pooled samples

## Discussion

Our findings revealed that false positivity rate of Cuffdiff2 and false negativity rates of DESeq2 and TSPM were high. Contrary to previous studies that supported the validity of RNA sample pooling for microarray based analyses of gene expression [21, 26], we documented the pooling bias in estimating differential gene expression, and high false positivity rate to detect DEGs for RNA-seq experiments employing pooling of low amount RNA samples from brain micro-punches. Our results corroborated previous studies, which indicated low sensitivity of DESeq [5, 27], high false positivity of Cuffdiff [4], and high sensitivity of edgeR [5]. False positivity and false negativity rates of TSPM have been reported to be dependent on the number of replicates [5, 6, 15]. This study did not evaluate the issues concerning read alignment [7], read counting [27], transcript assembly [8], and many novel DEG analysis methods [5, 28]. It included RNA samples, expected to be highly variable on their gene expression profiles, since amygdalae comprise multiple functionally distinct nuclei [29], and micro-punching of such regions in mouse brain is inherently imprecise.

Differences between the DEG analysis methods begin with their normalisation procedures [9]. edgeR uses a model, which incorporates normalisation factors as offsets that are estimated by trimmed mean of M values for each contig [30]. DESeq2 employs a relative log expression method [27]. Normalisation procedures for Cuffdiff2 consider total number of reads, gene length, variability within and between the conditions, and differential isoform expression [12, 18]. TSPM can accommodate various normalisation procedures, but works without normalisation by default [15]. It assumes Poisson distribution for the genes that are not over-dispersed. edgeR and DESeq2 model negative binomial distribution, while Cuffdiff2 follow beta negative binomial model to accommodate ambiguously mapped reads [12]. Principal source of variability between these methods is their dispersion estimation procedures [27]. DESeq2 is stringent to detect outliers and excludes genes with extreme read counts by default [31]. It considers the maximum a posteriori dispersion estimates, while edgeR moderates its dispersion estimates by their dispersion-mean relationship [32]. Cuffdiff2 includes covariances between different isoforms [12]. TSPM differs by its per-gene dispersion estimation without considering the information across genes [15]. Several unique correction procedures, such as multi-read correction, bias correction and effective length correction, are incorporated only in Cufflinks2 and Cuffdiff2 [33]. edgeR, DESeq2 and Cuffdiff2 calculate their p values by the generalized linear model (GLM) likelihood ratio test [32], GLM Wald test [31] and t-test [12], respectively. TSPM employs quasi or standard likelihood ratio tests, based on whether a gene is over-dispersed or not. Cuffdiff is more likely to estimate false positive statistically significant p values, when the gene expression is detected in only one group [4].

Our results favour the use of edgeR, among the four investigated methods, and discourage using RNA pooling in future RNA-seq experiments. Pooled samples do not represent the population variations in gene expression levels, and they cannot estimate within population variation [21]. Within-group variances of the pooled samples are less than true within-group variances of the individual samples. This leads to erroneously long DEG lists with low positive predictive values that limit practical use. If researchers plan RNA pooling because of saving costs or of limited starting material, stringent false discovery corrections and high-throughput validation of as many identified DEGs as possible should be considered. If the validation targets are chosen by random sampling from the list of identified DEGs, false discovery rates can be estimated cost-effectively [34]. An increase in the number of biological replicates, added into each pool, may help to minimise the pooling bias in estimating differential gene expression. Increasing the number of replicates is more effective to improve the power to detect DEGs than increasing sequencing depth above 10 million reads per sample [4, 35]. Limiting sequencing depth to 10 million reads per sample can reduce the costs and can help the biologists to sequence more replicates. Heterogeneity of biological variance among RNA samples may be larger than the dispersion, estimated by edgeR [36], and most contemporary RNA-seq experiments have been estimated to be under-powered by their design [10]. Hence, reducing the number of replicates by pooling will decrease the power and the ability to estimate within population variation further, and will increase pooling bias as well as false discovery rates (FDR).

Although edgeR was the most sensitive among these four methods, it did not detect differential expression of *Brd1* that was genetically modified in these mice. Employing two or more DEG analysis methods in parallel can enhance the overall sensitivity to detect true-positive DEGs [37], but consequent rise in the FDR will further increase the need for high-throughput validation of identified DEGs. In a hypothetical RNA-seq experiment with 10000 expressed genes, 100 DEGs, 5 % FDR 80 % power, minimum average read count of 1, and maximum dispersion of 0.5, 99 replicates need to be sequenced to detect a DEG with two-fold differential expression [10]. Sample size requirement will be more, if a DEG has low expression, less differential expression, and high dispersion. Until such large RNA-seq experiments become a reality, we cannot overemphasise the need for combined use of more sensitive DEG analysis methods and of high-throughput validation of identified DEGs.

## Conclusions

Among the four investigated methods for RNA-seq differential expression analyses using brain micropunches, edgeR detected more true-positive DEGs with high specificity. Moreover, we demonstrated limited utility of sample pooling strategies for RNA-seq in our setup. Pooled samples identified DEGs with high false positivity rates and low positive predictive values. On the basis of our results, we conclude that combined use of more sensitive DEG analysis methods and high-throughput validation of identified DEGs is desired for future RNA-seq experiments.

## Methods

### RNA samples

RNA samples were obtained from female mice, heterozygous for a targeted deletion of exon 3–5 of the *Brd1* gene (*Brd1*^+/−^) on a congenic C57BL/6NTac background, and from their WT littermates. 8–10 weeks old *Brd1*^+/−^ (n = 8) and WT mice (n = 8) were sacrificed. Their brains were snap frozen in 2-methylbutane and sectioned (1 mm) coronally using a slicer matrix (Zivic Instruments, Pittsburgh, USA) at −20 °C. Amygdalae were identified [38] and punched by a needle (1 mm diameter). Total RNA was extracted using Maxwell-16 system and LEV simplyRNA Tissue Kit (Promega, Madison, USA). Quantity of RNA was measured by NanoDrop 1000 version 3.7.1 (Thermo Fisher Scientific, Waltham, MA, USA) and their quality was assessed using Agilent 2100 Bioanalyzer (Agilent technologies, SantaClara, USA). All animal procedures were approved by the Danish National Committee for Ethics in Animal Experimentation.

### RNA-seq analysis

16 individual RNA samples (264 ng RNA/sample; 8/group, mean RNA Integrity Number (RIN) 7.53 (SD 0.31) [39]), 4 pools (2/group) that combined three individual samples (88 ng RNA/sample; 264 ng/pool) and 4 pools (2/group) that combined eight individual samples (33 ng RNA/sample; 264 ng/pool) were included. Each pool of the two pools/group was prepared by pooling equal amounts of the same three or eight RNA samples [Additional file 10]. cDNA was synthesised from all 24 RNA samples using random hexamer primers and libraries were prepared by TruSeq RNA sample preparation kit (Illumina, San Diego, USA). RNA-seq (50 bp; single-end; minimum 10 million clean reads/sample) was performed using Illumina HiSeq2000 (Illumina, San Diego, USA).

### DEG analyses

Reads that passed quality control (more than 90 % bases had less than 1 % sequencing error; no ambiguous bases) were aligned to the mouse genome (Mus_musculus.GRCm.38.72) with corresponding gene model annotation (Mus_musculus.GRCm38.72.gtf) by TopHat 2.0.6 [16]. Overall read alignment rates were above 90 % for all libraries. Aligned reads were counted by HTSeq 0.5.4 with “intersection-nonempty” overlap resolution mode [17]. DEG analyses with edgeR 3.2.4 [13, 27], Cuffdiff 2.1.1 [12, 18], DESeq2 1.0.19 [27, 31] and TSPM [15] followed previously published protocols using default parameters (unless stated differently). edgeR employed generalized linear models with tag-wise dispersion. As Cuffdiff2 do not work with count matrix, Tophat2 aligned reads were assembled into transcripts using Cufflinks 2.1.1 [18] with quartile normalisation, bias correction, multi-read correction, and with reference gene model annotation (Mus_musculus.GRCm.38.72.gtf). After combining all transcripts by Cuffmerge 2.1.1 [18] with reference gene model annotation (Mus_musculus.GRCm.38.72.gtf), Cuffdiff2 identified DEGs with geometric library normalisation and per-condition dispersion estimation. Adjusted p values were calculated by Benjamini-Hochberg false discovery correction (5 %) for all methods. Genes with adjusted p values less than 0.05 were considered as DEGs. Codes for the analysis methods were provided in the Additional file 11.

### Validation of DEGs by qPCR

16 RNA samples were obtained by the procedures, described above, from another batch of female *Brd1*^+/−^ and WT mice (8/group). 180 ng total RNA was reverse transcribed by iScript Select cDNA Synthesis Kit (Bio-Rad, Hercules, USA). All eight DEGs, detected by DESeq2 and TSPM, were selected for validation. 107 more genes were randomly selected for validation from the list of remaining 191 DEGs, detected by Cuffdiff2 and edgeR. After 10–20 cycles of specific target amplification with PreAmp master mix (Fluidigm, San Francisco, USA), high-throughput qPCR was performed on the BioMark HD (Fluidigm, San Francisco, USA), using 48.48 dynamic arrays (Fluidigm, San Francisco, USA) and SsoFast EvaGreen Low ROX kit (Bio-Rad, Hercules, USA) [Additional files 12 & 13]. A DEG, detected by the RNA-seq DEG analysis methods, was considered as a true-positive DEG, if it satisfied the following criteria, (i) Both RNA-seq and qPCR showed same direction (upregulation or down-regulation) of differential expression, (ii) Differential expression fold change, estimated by qPCR, was either above 1.25 or below 0.80 (LFC cut-off was ±0.3219) [34]. Spearman correlation coefficients, root-mean-square deviations and kappa statistics were calculated using STATA 13.1 (StataCorp LP, Texas, USA).

### Availability of supporting data

Digital expression count matrix of our RNA-seq data is available as the Additional date file 1 with the online version of this paper.

## Additional files

Additional file 1:
**A digital expression count matrix of RNA-seq data from amygdalae of female** ***Brd1*** ^**+/−**^ **mice (n = 8) and female wild-type (WT) littermates (n = 8).**
Additional file 2:
**A table presenting Cuffdiff2 analyses of RNA-seq data from amygdalae of female** ***Brd1*** ^**+/−**^ **mice (n = 8) and female WT littermates (n = 8).**
Additional file 3:
**A table presenting DESeq2 analyses of RNA-seq data from amygdalae of female** ***Brd1*** ^**+/−**^ **mice (n = 8) and female WT littermates (n = 8).**
Additional file 4:
**A table presenting TSPM analyses of RNA-seq data from amygdalae of female** ***Brd1*** ^**+/−**^ **mice (n = 8) and female WT littermates (n = 8).**
Additional file 5:
**Presents the results of Reverse-transcription quantitative Polymerase Chain Reaction (qPCR) validation of 115 differentially expressed genes (.xlsx file).** This workbook has three worksheets. Third worksheet presents relevant data labels. Additional file 6:
**A figure, “Comparisons of logarithmic fold changes (LFC), estimated by qPCR, with LFC, estimated by Cuffdiff2, DESeq2, edgeR, and TSPM” (.jpeg image).** (A) qPCR and Cuffdiff2; (B) qPCR and DESeq2; (C) qPCR and edgeR; (D) qPCR and TSPM. Additional file 7:
**Presents the results of edgeR analyses of RNA-seq data from 8-sample as well as 3-sample pools of RNA and from three corresponding individual samples (.xlsx file).** This workbook has three worksheets. First worksheet is a table presenting edgeR analyses of RNA-seq data of 8-sample pools of RNA from amygdalae of female *Brd1* ^+/−^ mice (n = 2) and female WT littermates (n = 2). Second worksheet is a table presenting edgeR analyses of RNA-seq data of 3-sample pools of RNA from amygdalae of female *Brd1* ^+/−^ mice (n = 2) and female WT littermates (n = 2). Third worksheet is a table presenting edgeR analyses of RNA-seq data of three corresponding individual RNA samples from amygdalae of female *Brd1* ^+/−^ mice (n = 3) and female WT littermates (n = 3). Additional file 8:
**Presents the results of Cuffdiff2 analyses of RNA-seq data from 8-sample as well as 3-sample pools of RNA and from three corresponding individual samples (.xlsx file).** This workbook has three worksheets. First worksheet is a table presenting Cuffdiff2 analyses of RNA-seq data of 8-sample pools of RNA from amygdalae of female *Brd1* ^+/−^ mice (n = 2) and female WT littermates (n = 2). Second worksheet is a table presenting Cuffdiff2 analyses of RNA-seq data of 3-sample pools of RNA from amygdalae of female *Brd1* ^+/−^ mice (n = 2) and female WT littermates (n = 2). Third worksheet is a table presenting Cuffdiff2 analyses of RNA-seq data of three corresponding individual RNA samples from amygdalae of female *Brd1* ^+/−^ mice (n = 3) and female WT littermates (n = 3). Additional file 9:
**A figure, “Agreement between Cuffdiff2 analyses of pooled RNA samples and of corresponding individual samples of RNA”.** (A & C) Intersections between the DEGs, detected by Cuffdiff2, in RNA-seq data from pooled RNA (two pools/ group) and of data from three (A) or eight (C) corresponding individual samples of RNA; Rectangle represents all expressed genes: (A) Three RNA samples/pool; (C) Eight RNA samples/pool, (B & D) Correlation between the logarithmic (base 2) fold changes (LFC) in expression that were estimated by sequencing RNA-pools (two pools/group) and by sequencing three (B) or eight (D) corresponding individual samples of RNA: (B) Three RNA samples/ pool; (D) Eight RNA samples/pool. Additional file 10:
**A figure, “RNA-seq analysis of 16 individual and 8 pooled RNA samples” (.jpeg image).** It presents the details of our pooling strategy. Additional file 11:
**Presents the codes and the Galaxy web tools for DESeq2, TSPM, edgeR, and Cuffdiff2.**
Additional file 12:
**A table presenting overview of RNA extraction, cDNA synthesis, specific target amplification and high-throughput qPCR procedures.**
Additional file 13:
**A table presenting forward and reverse primer sequences for qPCR validation of 115 differentially expressed and 7 normalising (reference) genes (.xlsx file).** This workbook has two worksheets.
