# Supplementary figures and images for: Experimental validation of methods for differential gene expression analysis and sample pooling in RNA-seq
Source: BMC Genomics. 2015 Jul 25;16(1):548. doi: 10.1186/s12864-015-1767-y (PMC4515013; doi:10.1186/s12864-015-1767-y)

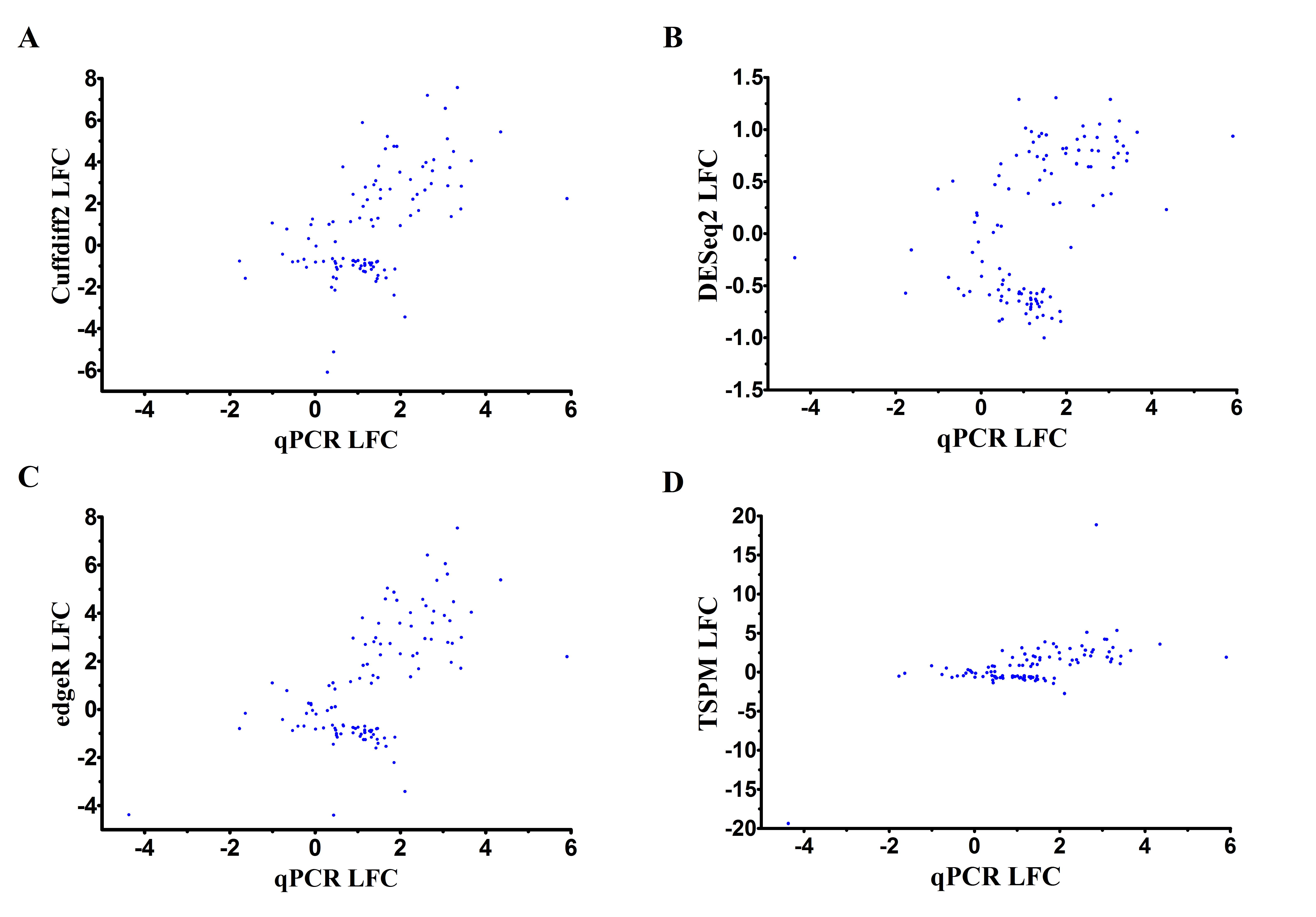

Supplement: Additional file 6: — A figure, “Comparisons of logarithmic fold changes (LFC), estimated by qPCR, with LFC, estimated by Cuffdiff2, DESeq2, edgeR, and TSPM” (.jpeg image). (A) qPCR and Cuffdiff2; (B) qPCR and DESeq2; (C) qPCR and edgeR; (D) qPCR and TSPM. [file 12864_2015_1767_MOESM6_ESM.jpeg]

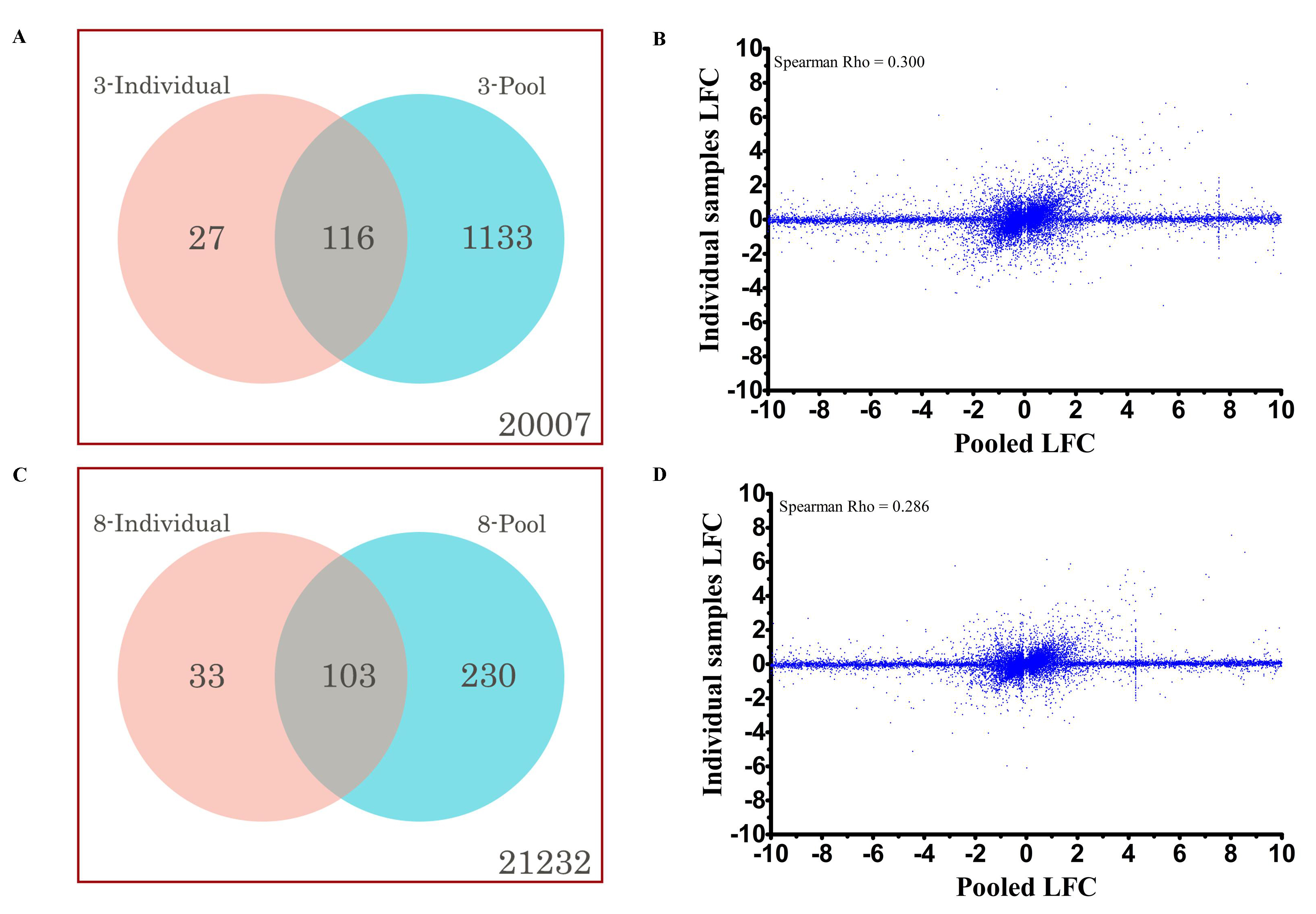

Supplement: Additional file 9: — A figure, “Agreement between Cuffdiff2 analyses of pooled RNA samples and of corresponding individual samples of RNA”. (A & C) Intersections between the DEGs, detected by Cuffdiff2, in RNA-seq data from pooled RNA (two pools/ group) and of data from three (A) or eight (C) corresponding individual samples of RNA; Rectangle represents all expressed genes: (A) Three RNA samples/pool; (C) Eight RNA samples/pool, (B & D) Correlation between the logarithmic (base 2) fold changes (LFC) in expression that were estimated by sequencing RNA-pools (two pools/group) and by sequencing three (B) or eight (D) corresponding individual samples of RNA: (B) Three RNA samples/ pool; (D) Eight RNA samples/pool. [file 12864_2015_1767_MOESM9_ESM.jpeg]

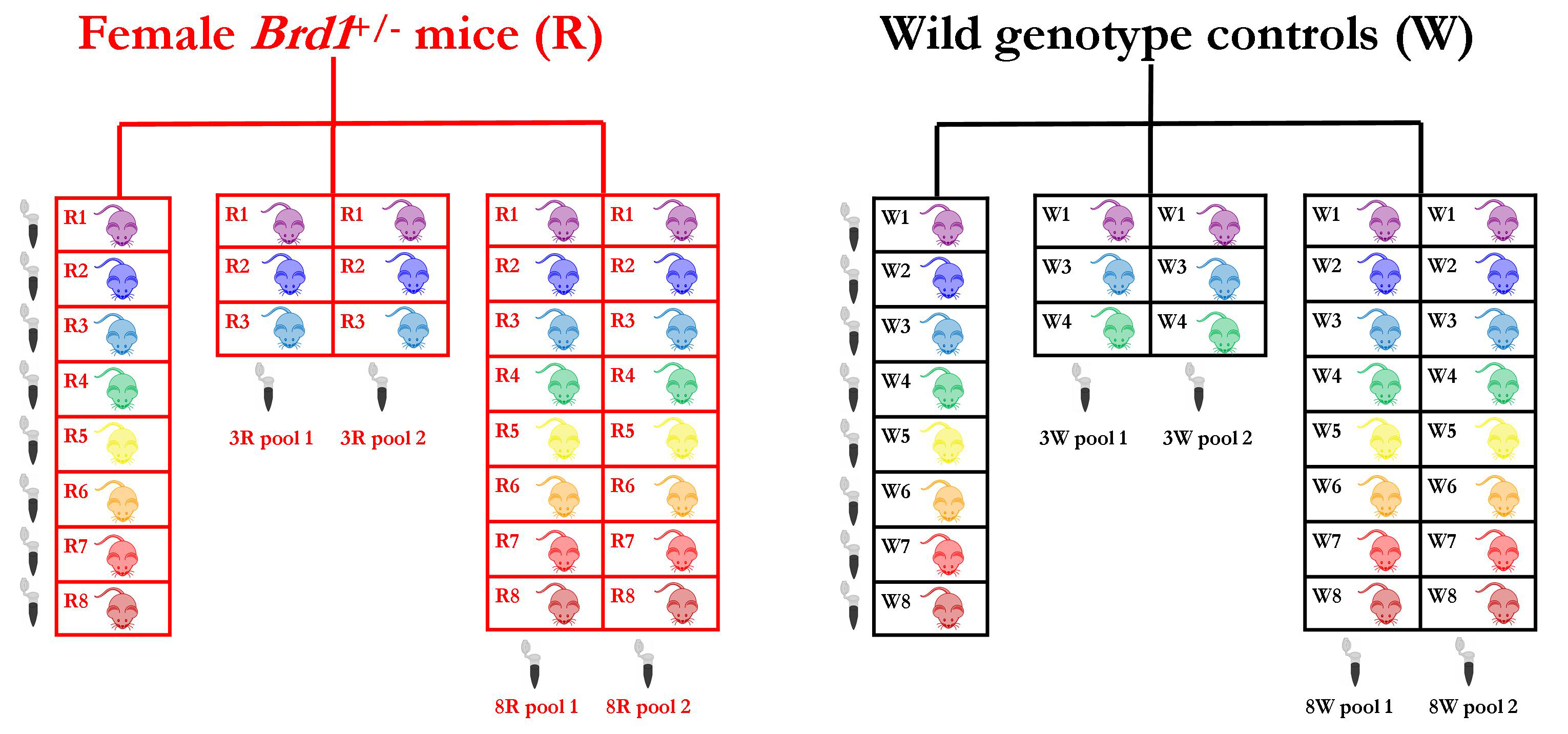

Supplement: Additional file 10: — A figure, “RNA-seq analysis of 16 individual and 8 pooled RNA samples” (.jpeg image). It presents the details of our pooling strategy. [file 12864_2015_1767_MOESM10_ESM.jpeg]
