# Supplementary material for: Experimental validation of methods for differential gene expression analysis and sample pooling in RNA-seq
Source: BMC Genomics. 2015 Jul 25;16(1):548. doi: 10.1186/s12864-015-1767-y (PMC4515013; doi:10.1186/s12864-015-1767-y)
Supplement: Additional file 11: — Presents the codes and the Galaxy web tools for DESeq2, TSPM, edgeR, and Cuffdiff2. [file 12864_2015_1767_MOESM11_ESM.pdf]

```r
setwd("/Volumes/fles$/RNAseqAnto/DESeqAnalysis/")
ensembl = useMart("ensembl",dataset="mmusculus_gene_ensembl")
```

1 HTSeq counting

In HTSeq the counting is done per item, i.e. counting directly the number of reads overlapping a feature. In this case features are passed through a file: this analysis uses the GTF file provided with the .bam files.

The specificity of HTSeq is the capability to control the exclusion criteria of the counting, as explained in the following picture.

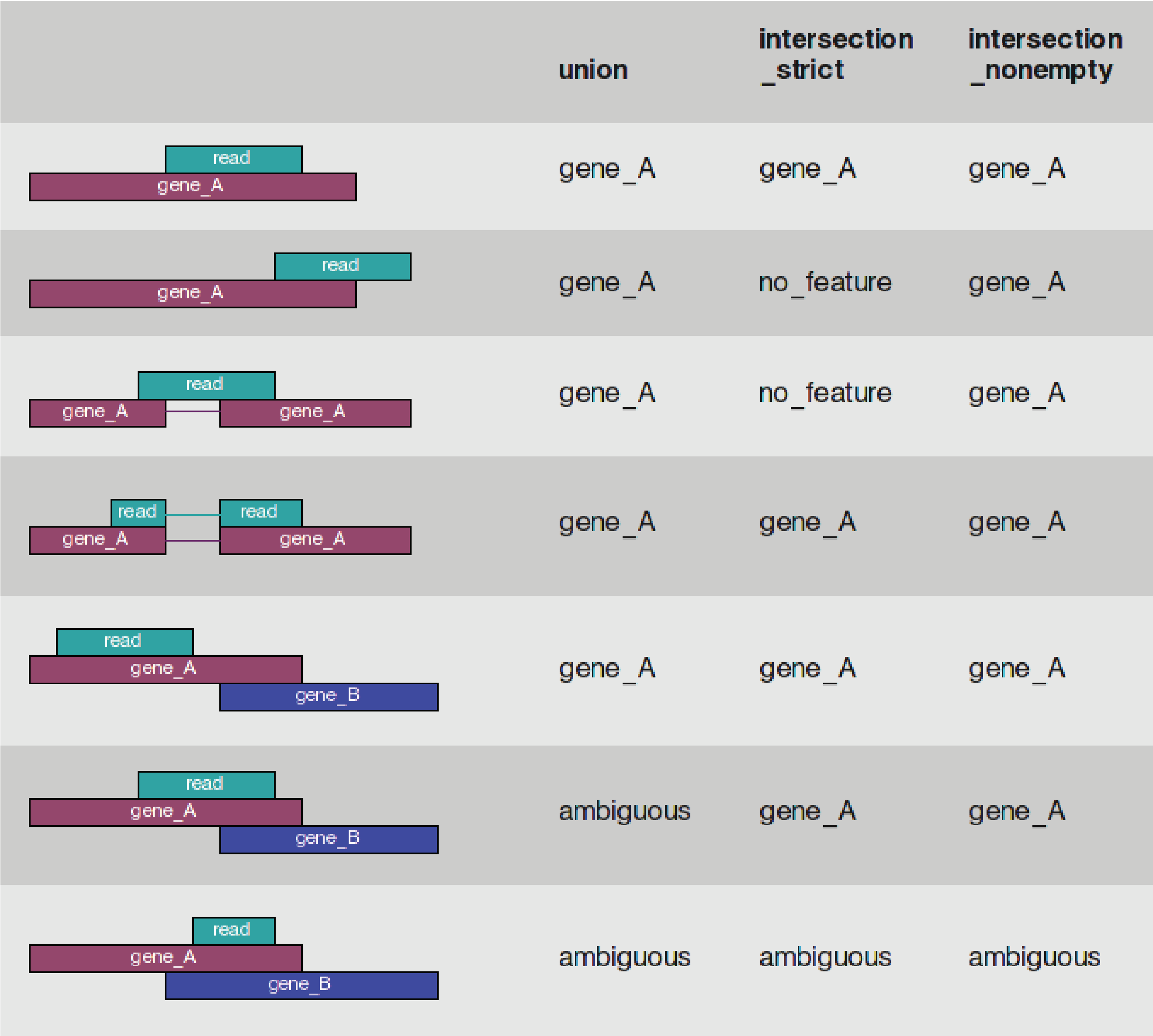

Figure 1: Different modes of read counting by HTSeq - figure from http://www-huber.embl.de/users/anders/HTSeq

2 DESeq2 Analysis

The analysis with DESeq2 has been carried out using counts performed with HTTSeq (python script). The counts have been performed using the "intersection non empty" method, as detailed in the previous figure.

2.1 HTSeq counts - Intersection

In the following we first prepare the data (sample names and condition) and then import the counts

```
setwd("/Volumes/fles$/RNAseqAnto/BAM files/")
sampleFiles <- grep("noempty",dir(),value=TRUE)
sampleNames <- gsub(".bam.HTSeq.noempty", "", sampleFiles)
sampleCondition <- c(rep("knockout",8),rep("wildtype",8))
sampleTable <- data.frame(sampleName = sampleNames,
                          fileName = sampleFiles,
                          condition = sampleCondition)
ddsHTSeq.int <- DESeqDataSetFromHTSeqCount(sampleTable = sampleTable,
                                       directory = "/Volumes/fles$/RNAseqAnto/BAM files/",
                                       design= ~ condition)
colData(ddsHTSeq.int)$condition <- factor(colData(ddsHTSeq.int)$condition,
                                      levels=c("wildtype","knockout"))
setwd("/Volumes/fles$/RNAseqAnto/DESeqAnalysis")
save.image("rev_brd1RNASeq.RData")
```

Then we perform the actual calculation.
In particular, as described in **DESeq2** the function performs a default analysis through the steps:

1. estimation of size factors: estimateSizeFactors
2. estimation of dispersion: estimateDispersions
3. Negative Binomial GLM fitting and Wald statistics: nbinomWaldTest

and as far as the dispersion is concerned, the authors clarify as follows: "The fitting proceeds as follows: for each gene, an estimate of the dispersion is found which maximizes the Cox Reid-adjusted profile likelihood (the methods of Cox Reid-adjusted profile likelihood maximization for estimation of dispersion in RNA-Seq data were developed by McCarthy, et al. (2012), first implemented in the edgeR package in 2010); a trend line capturing the dispersion-mean relationship is fit to the maximum likelihood estimates; a normal prior is determined for the log dispersion estimates centered on the predicted value from the trended fit with variance equal to the difference between the observed variance of the log dispersion estimates and the expected sampling variance; **finally maximum a posteriori dispersion estimates are returned.** This final dispersion parameter is used in subsequent tests."

```
ddsHTSeq.int <- DESeq(ddsHTSeq.int)
resplot <- results(ddsHTSeq.int, addMLE=TRUE)
HTSeqIntRes <- results(ddsHTSeq.int)
HTSeqIntRes <- as.data.frame(HTSeqIntRes)
HTSeqIntRes$geneID <- row.names(HTSeqIntRes)
```

The data are annotated using Biomart:

```
HTSeqIntRes <- merge(
  HTSeqIntRes,
  getBM(filters=c("ensembl_gene_id"),
        attributes=c("ensembl_gene_id", "wikigene_name"),
        values=HTSeqIntRes$geneID, mart=ensembl),
  by.x="geneID",
```

```
  by.y="ensembl_gene_id"
  )
HTSeqIntRes <-HTSeqIntRes[order(HTSeqIntRes$padj),]
write.table(HTSeqIntRes, file="_rev_dseq_HTScounts_intersection_results.txt",
            sep="\t", quote=F, row.names=F)
save.image("rev_brd1RNASeq.RData")
```

The MA plot of the results:

```
plotMA(resplot, main="DESeq2", ylim=c(-2,2))
```

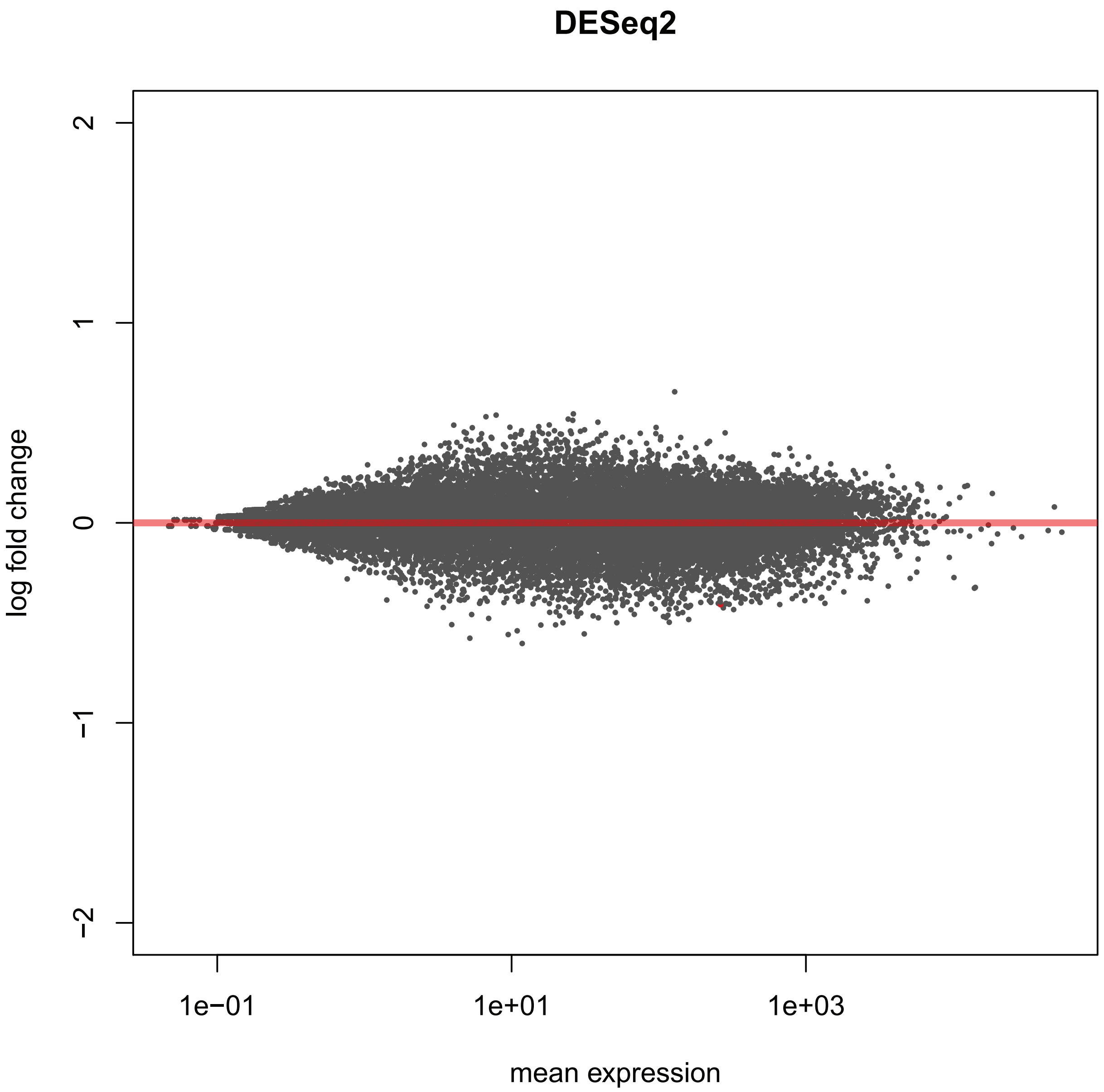

Figure 2: Log2 fold changes over the mean of normalised counts, in HTSeq with the Intersection method

And the table of results:

```
htseqint.table<-xtable(HTSeqIntRes[c(1:30),],
                       display=c("s","s","f","f","f","f","g","g","s"))
print(htseqint.table,tabular.environment='longtable',include.rownames = FALSE)
```

geneID	baseMean	log2FoldChange	lfcSE	stat	pvalue	padj	wikigene_name
ENSMUSG00000022387	262.50	-0.41	0.06	-6.74	1.6e-11	4.1e-07	Brd1
ENSMUSG00000016918	128.30	0.66	0.17	3.92	8.7e-05	0.54	Sulf1
ENSMUSG00000052861	26.29	0.54	0.14	3.96	7.4e-05	0.54	Dnah6
ENSMUSG00000053153	7.85	0.54	0.14	3.95	7.7e-05	0.54	Spag16
ENSMUSG00000000028	6.83	0.08	0.16	0.47	0.64	0.96	Cdc45
ENSMUSG00000000037	13.69	-0.11	0.16	-0.65	0.52	0.96	Scml2
ENSMUSG00000000056	308.58	-0.09	0.10	-0.91	0.36	0.96	Narf
ENSMUSG00000000078	169.45	0.10	0.14	0.72	0.47	0.96	Klf6
ENSMUSG00000000088	447.25	-0.12	0.10	-1.18	0.24	0.96	Cox5a
ENSMUSG00000000093	19.35	-0.16	0.16	-1.06	0.29	0.96	Tbx2
ENSMUSG00000000094	2.00	0.23	0.13	1.74	0.082	0.96	Tbx4
ENSMUSG00000000120	103.91	-0.09	0.11	-0.82	0.41	0.96	Ngfr
ENSMUSG00000000125	16.54	-0.33	0.16	-2.02	0.044	0.96	Wnt3
ENSMUSG00000000126	33.82	0.08	0.11	0.70	0.49	0.96	Wnt9a
ENSMUSG00000000127	79.23	0.06	0.11	0.58	0.56	0.96	Fert2
ENSMUSG00000000131	649.71	-0.12	0.07	-1.76	0.079	0.96	Xpo6
ENSMUSG00000000134	244.85	0.04	0.08	0.53	0.6	0.96	Tfe3
ENSMUSG00000000142	160.13	0.14	0.12	1.20	0.23	0.96	Axin2
ENSMUSG00000000148	144.62	0.15	0.11	1.39	0.16	0.96	Brat1
ENSMUSG00000000149	655.17	-0.34	0.15	-2.22	0.026	0.96	Gna12
ENSMUSG00000000157	0.74	0.17	0.10	1.75	0.079	0.96	Itgb2l
ENSMUSG00000000167	9.72	0.30	0.16	1.82	0.069	0.96	Pih1d2
ENSMUSG00000000168	502.64	-0.07	0.07	-0.96	0.34	0.96	Dlat
ENSMUSG00000000171	301.05	-0.09	0.09	-0.99	0.32	0.96	Sdhd
ENSMUSG00000000184	370.03	0.27	0.16	1.64	0.1	0.96	Ccnd2
ENSMUSG00000000194	334.70	-0.04	0.06	-0.69	0.49	0.96	Gpr107
ENSMUSG00000000202	94.58	-0.12	0.14	-0.85	0.4	0.96	Btbd17
ENSMUSG00000000214	39.43	-0.17	0.13	-1.24	0.22	0.96	Th
ENSMUSG00000000215	0.17	0.04	0.04	1.04	0.3	0.96	Ins2
ENSMUSG00000000223	571.25	0.12	0.14	0.84	0.4	0.96	Drp2

```r
sessionInfo()

## R version 3.1.2 (2014-10-31)
## Platform: x86_64-apple-darwin13.4.0 (64-bit)
##
## locale:
## [1] en_GB.UTF-8/en_GB.UTF-8/en_GB.UTF-8/C/en_GB.UTF-8/en_GB.UTF-8
##
## attached base packages:
## [1] parallel  stats4    stats     graphics  grDevices utils     datasets  methods
## [9] base
##
## other attached packages:
##  [1] biomaRt_2.22.0           xtable_1.7-4             gplots_2.15.0
##  [4] RColorBrewer_1.1-2       DESeq2_1.6.2             RcppArmadillo_0.4.550.1.0
##  [7] Rcpp_0.11.3              GenomicRanges_1.18.3     GenomeInfoDb_1.2.3
## [10] IRanges_2.0.1            S4Vectors_0.4.0          BiocGenerics_0.12.1
## [13] knitr_1.8
##
## loaded via a namespace (and not attached):
##  [1] acepack_1.3-3.3      annotate_1.44.0      AnnotationDbi_1.28.1 base64enc_0.1-2
##  [5] BatchJobs_1.5        BBmisc_1.8           Biobase_2.26.0       BiocParallel_1.0.0
##  [9] BiocStyle_1.4.1      bitops_1.0-6         brew_1.0-6           caTools_1.17.1
## [13] checkmate_1.5.1      cluster_1.15.3       codetools_0.2-9      colorspace_1.2-4
## [17] DBI_0.3.1            digest_0.6.6         evaluate_0.5.5       fail_1.2
## [21] foreach_1.4.2        foreign_0.8-61       formatR_1.0          Formula_1.1-2
## [25] gdata_2.13.3         genefilter_1.48.1    geneplotter_1.44.0   ggplot2_1.0.0
## [29] grid_3.1.2           gtable_0.1.2         gtools_3.4.1         highr_0.4
## [33] Hmisc_3.14-6         iterators_1.0.7      KernSmooth_2.23-13   lattice_0.20-29
## [37] latticeExtra_0.6-26  locfit_1.5-9.1       MASS_7.3-35          munsell_0.4.2
## [41] nnet_7.3-8           plyr_1.8.1           proto_0.3-10         RCurl_1.95-4.5
## [45] reshape2_1.4.1       rpart_4.1-8          RSQLite_1.0.0        scales_0.2.4
## [49] sendmailR_1.2-1      splines_3.1.2        stringr_0.6.2        survival_2.37-7
## [53] tools_3.1.2          XML_3.98-1.1         XVector_0.6.0
```

3 TSPM analysis

We present the TSPM.R codes for getting log fold change, p-value, and adjusted p-values below. Aligned RNA-Seq reads were counted by HTSeq 0.5.4 with "intersection-nonempty" overlap resolution mode.

In.xls: a matrix of RNA-Seq read counts. The value in the ith row and the jth column of the matrix indicates the number of reads that have been mapped to gene i in the sample j

x1: a vector of treatment group factors indicates the sample in corresponding column of In.xls comes from specific group.

```
source("D:\\TSPM.R")
countTable<-read.delim("D:\\In.xls",header=TRUE,stringsAsFactors=TRUE, row.names="GeneID")
x1<-c("B","B","B","B","B","B","B","B","A","A","A","A","A","A","A","A")
x0<- rep(1, times=length(x1))
lib.size <- apply(countTable,2,sum)
result <- TSPM(countTable, x1, x0, lib.size)
write.table(as.data.frame(result),"D:\\TSPM.Result.xls",sep="\t",row.names =FALSE)
```

Codes in TSPM.R which were modified for our need are marked in blue.

```
#-------------------------------------------------------------------
##   Name: TSPM.R
##        R code for the paper by Paul L. Auer and R.W. Doerge:
##   "A Two-Stage Poisson Model for Testing RNA-Seq Data"
##   Date: February 2011
##   Contact: Paul Auer          plivermo@fhcrc.org
##            R.W. Doerge        doerge@purdue.edu

##   Example:
##   counts <- matrix(0, nrow=1000, ncol=10)
##   for(i in 1:1000){
##        lambda <- rpois(n=1, lambda=10)
##        counts[i,] <- rpois(n=10, lambda=lambda)
```

```
##   }
##   x1 <- gl(n=2, k=5, labels=c("T", "C"))
##   x0 <- rep(1, times=10)
##   lib.size <- apply(counts,2,sum)
##   result <- TSPM(counts, x1, x0, lib.size)
##-------------------------------------------------------------------

###########################################################################
###### The TSPM function ##################################################
###########################################################################

TSPM <- function(counts, x1, x0, lib.size, alpha.wh=0.05){

## Input:
#counts:      a matrix of RNA-Seq gene counts (genes are rows, samples are columns)
#x1:          a vector of treatment group factors (under the alternative hypothesis)
#x0:          a vector of treatment group factors (under the null hypothesis)
#lib.size:    a vector of RNA-Seq library sizes. This could simply be obtained
#                 by specifying lib.size <- apply(counts,2,sum). It may also be any other
#                 appropriate scaling factor.
#alpha.wh:    the significance threshold to use for deciding whether a gene is overdispersed.
#                    Defaults to 0.05.

## Output:
#log.fold.change:       a vector containing the estimated log fold changes for each gene
#pvalues:               a vector containing the raw p-values testing differential expression for each
gene.
#index.over.disp:       a vector of integer values containing the indices of the over-dispersed
genes.
#index.not.over.disp: a vector of integer values containing the indices of the non-over-dispersed
genes.
```

```
#padj:              a vector containing the p-values after adjusting for multiple testing using the
#                   method of Benjamini-Hochberg

######## The main loop that fits the GLMs to each gene ########################

### Initializing model parameters ####
n <- dim(counts)[1]
per.gene.disp <- NULL
LRT <- NULL
score.test <- NULL
LFC <- NULL

###### Fitting the GLMs for each gene #################
    for(i in 1:n){
        ### Fit full and reduced models ###
        model.1 <- glm(as.numeric(counts[i,]) ~ x1, offset=log(lib.size), family=poisson)
        model.0 <- glm(as.numeric(counts[i,]) ~ x0, offset=log(lib.size), family=poisson)

        ### Obtain diagonals of Hat matrix from the full model fit ###
        hats <- hatvalues(model.1)

        ### Obtain Pearson overdispersion estimate ####
        per.gene.disp[i] <- sum(residuals(model.1, type="pearson")^2)/model.1$df.residual

        ### Obtain Likelihood ratio statistic ####
        LRT[i] <- deviance(model.0)-deviance(model.1)

        ### Obtain score test statistic ####
        score.test[i] <- 1/(2*length(counts[i,])) * sum(residuals(model.1, type="pearson")^2 -
((counts[i,] - hats*model.1$fitted.values)/model.1$fitted.values))^2
```

```
        ### Obtain the estimated log fold change ###
        #LFC[i] <- -model.1$coef[2]
         LFC[i] <- model.1$coef[2]
    }

## Initialize parameters for Working-Hotelling bands around the score TSs ###
qchi <- qchisq(df=1, (1:n-0.5)/n)
MSE <- 2
UL <- NULL

#### Obtain the upper boundary of the WH bands
###########################################
xbar <- mean(qchi)
bottom <- sum((qchi-xbar)^2)
top <- (qchi-xbar)^2
s <- sqrt(MSE*(1/n) + (top/bottom))
W <- sqrt(2*qf(df1=1, df2=n-1, p=1-(alpha.wh/n)))
UL <- pmax(qchi + W*s,1)

###### Obtain the indices of the over-dispersed and not-over-dispersed genes, respectively
##########

cutoff <- min(which(sort(score.test)-UL > 0))
temp <- cutoff-1 + seq(cutoff:length(score.test))
over.disp <- which(score.test %in% sort(score.test)[temp])
not.over.disp <- setdiff(1:length(score.test), over.disp)

###### Compute p-values ########################################
p.f <- pf(LRT[over.disp]/per.gene.disp[over.disp], df1=1, df2=model.1$df.residual,
lower.tail=FALSE)
p.chi <- pchisq(LRT[not.over.disp], df=1, lower.tail=FALSE)
```

```
p <- NULL
p[over.disp] <- p.f
p[not.over.disp] <- p.chi

##### Adjust the p-values using the B-H method ####################
p.bh.f <- p.adjust(p.f, method="BH")
p.bh.chi <- p.adjust(p.chi, method="BH")
final.p.bh.tagwise <- NULL
final.p.bh.tagwise[over.disp] <- p.bh.f
final.p.bh.tagwise[not.over.disp] <- p.bh.chi

#####output with over.disp#####################
index<-NULL
index[over.disp]<-rep("Y",times=length(over.disp))
index[not.over.disp]<-rep("N",times=length(not.over.disp))

### Output ###
#list(log.fold.change=LFC, pvalues=p, index.over.disp=over.disp,
index.not.over.disp=not.over.disp,padj=final.p.bh.tagwise)
list(log.fold.change=LFC, pvalues=p, padj=final.p.bh.tagwise,overdispersion=index)
}
```

4 edgeR Analysis

We used the same HTSeq generated count matrix as described earlier as the input for analysis with edgeR v3.2.4 from BioConductor. The current version of Differential Count Models tool in August 2103 from the Galaxy Tool Shed was used to automate the analysis. This is available for automated installation into any Galaxy instance through the administrative interface to the test Tool Shed or directly at https://testtoolshed.g2.bx.psu.edu/view/fubar/differential_count_models. Parameter settings, sessionInfo() output and code generated by the Galaxy tool are shown below.

edgeR parameter settings recorded with the Galaxy job:

Tool: Differential_Count

Name:	AarhusmousedataDGE_topTable_edgeR.xls
Created:	Fri Aug 23 13:05:28 2013 (UTC)
Filesize:	7.7 MB
Format:	tabular
Galaxy Tool Version:	0.20
Tool Standard Output:	stdout
Tool Standard Error:	stderr
Tool Exit Code:	0
API ID:	dc3a498774df8e67
Full Path:	/data/extended/galaxy_data_store/files/037/dataset_37281.dat
Job Command-Line:	python /data/extended/galaxy/tools/rgedgeR/rgToolFactory.py --script_path "/data/extended/galaxy_data_store/job_working_directory/029/29194/tmpQ9orJX" --interpreter "Rscript" --tool_name "DifferentialCounts" --output_dir "/data/extended/galaxy_data_store/job_working_directory/029/29194/dataset_37282_files" --output_html "/data/extended/galaxy_data_store/job_working_directory/029/29194/galaxy_dataset_37282.dat" --make_HTML "yes"

Input Parameter	Value	Note for rerun
Select an input matrix - rows are contigs, columns are counts for each sample	1: Digital Expression Matrix BRD1 Amygdala.xls	
Title for job outputs	Aarhus mouse data DGE	
Treatment Name	R	
Select columns containing treatment.	10 11 12 13 14 15 16 17	
Control Name	WT	
Select columns containing control.	2 3 4 5 6 7 8 9	
IF SUBJECTS NOT ALL INDEPENDENT! Enter comma separated strings to indicate sample labels for (eg) pairing - must be one for every column in input		
Non-differential contig count quantile threshold - zero to analyze all non-zero read count contigs	0.3	
Non differential filter - remove contigs below a threshold (1 per million) for half or more samples	False	
Run this model using edgeR	T	
prior.df for tagwise dispersion - larger value = more squeezing of tag dispersions to common dispersion. Replaces prior.n and prior.df = prior.n * residual.df	20	
Run the same model with DESeq2 and compare findings	F	
Run the same model with Voom/limma and compare findings	Do not run VOOM	
P value threshold for FDR filtering for amily wise error rate control	0.05	
FDR (Type II error) control method	fdr	

Output from sessionInfo() at the end of script:

R version 3.0.1 (2013-05-16)

Platform: x86_64-unknown-linux-gnu (64-bit)

locale:

```
[1] LC_CTYPE=en_AU.UTF-8       LC_NUMERIC=C               LC_TIME=en_AU.UTF-8
LC_COLLATE=en_AU.UTF-8     LC_MONETARY=en_AU.UTF-8
LC_MESSAGES=en_AU.UTF-8    LC_PAPER=C                 LC_NAME=C
LC_ADDRESS=C               LC_TELEPHONE=C
LC_MEASUREMENT=en_AU.UTF-8 LC_IDENTIFICATION=C
```

attached base packages:

```
 [1] parallel  splines   methods   grid      stats     graphics  grDevices utils     datasets  base
```

other attached packages:

```
 [1] RColorBrewer_1.0-5      DESeq2_1.0.19           RcppArmadillo_0.3.910.0 Rcpp_0.10.4
lattice_0.20-23         Biobase_2.20.1          GenomicRanges_1.12.4    IRanges_1.18.3
BiocGenerics_0.6.0      edgeR_3.2.4             limma_3.16.7            gplots_2.11.3
MASS_7.3-28             KernSmooth_2.23-10      caTools_1.14            gdata_2.13.2
gtools_3.0.0            stringr_0.6.2
```

loaded via a namespace (and not attached):

```
 [1] annotate_1.38.0      AnnotationDbi_1.22.6 bitops_1.0-6         DBI_0.2-7
genefilter_1.42.0    locfit_1.5-9.1       RSQLite_0.11.4       stats4_3.0.1         survival_2.37-4
XML_3.98-1.1         xtable_1.7-1
```

edgeR Code generated and run in Galaxy:

```
# edgeR.Rscript

# updated npv 2011 for R 2.14.0 and edgeR 2.4.0 by ross

# Performs DGE on a count table containing n replicates of two conditions

# Parameters

# 1 - Output Dir

# Original edgeR code by: S.Lunke and A.Kaspi

reallybig = log10(.Machine$double.xmax)

reallysmall = log10(.Machine$double.xmin)

library('stringr')

library('gplots')

library('edgeR')

hmap2 = function(cmat,nsamp=100,outpdfname='heatmap2.pdf',
```

```
TName='Treatment',group=NA,myTitle='title goes here')

{

# Perform clustering for significant pvalues after controlling FWER

    samples = colnames(cmat)

    gu = unique(group)

    gn = rownames(cmat)

    if (length(gu) == 2) {

        col.map = function(g) {if (g==gu[1]) "#FF0000" else "#0000FF"}

        pcols = unlist(lapply(group,col.map))

        } else {

        colours = rainbow(length(gu),start=0,end=4/6)

        pcols = colours[match(group,gu)]        }

    dm = cmat[(! is.na(gn)),]

    # remove unlabelled hm rows

    nprobes = nrow(dm)

    # sub = paste('Showing',nprobes,'contigs ranked for evidence of differential abundance')

    if (nprobes > nsamp) {

      dm =dm[1:nsamp,]

      #sub = paste('Showing',nsamp,'contigs ranked for evidence for differential abundance out
of',nprobes,'total')

    }

    newcolnames = substr(colnames(dm),1,20)

    colnames(dm) = newcolnames

    pdf(outpdfname)

heatmap.2(dm,main=myTitle,ColSideColors=pcols,col=topo.colors(100),dendrogram="col",
key=T,density.info='none',
```

```
        Rowv=F,scale='row',trace='none',margins=c(8,8),cexRow=0.4,cexCol=0.5)

    dev.off()

}

hmap =
function(cmat,nmeans=4,outpdfname="heatMap.pdf",nsamp=250,TName='Treatment',group
=NA,myTitle="Title goes here")

{

    # for 2 groups only was

    #col.map = function(g) {if (g==TName) "#FF0000" else "#0000FF"}

    #pcols = unlist(lapply(group,col.map))

    gu = unique(group)

    colours = rainbow(length(gu),start=0.3,end=0.6)

    pcols = colours[match(group,gu)]

    nrows = nrow(cmat)

    mtitle = paste(myTitle,'Heatmap: n contigs =',nrows)

    if (nrows > nsamp)  {

            cmat = cmat[c(1:nsamp),]

            mtitle = paste('Heatmap: Top ',nsamp,' DE contigs (of ',nrows,')',sep='')

        }

    newcolnames = substr(colnames(cmat),1,20)

    colnames(cmat) = newcolnames

    pdf(outpdfname)

heatmap(cmat,scale='row',main=mtitle,cexRow=0.3,cexCol=0.4,Rowv=NA,ColSideColors=
pcols)

    dev.off()

}
```

```
qqPlot = function(descr='qqplot',pvector, outpdf='qqplot.pdf',...)
# stolen from https://gist.github.com/703512
{
   o = -log10(sort(pvector,decreasing=F))
   e = -log10( 1:length(o)/length(o) )
   o[o==-Inf] = reallysmall
   o[o==Inf] = reallybig
   maint = descr
   pdf(outpdf)
   plot(e,o,pch=19,cex=1, main=maint, ...,
      xlab=expression(Expected~~-log[10](italic(p))),
      ylab=expression(Observed~~-log[10](italic(p))),
      xlim=c(0,max(e)), ylim=c(0,max(o)))
   lines(e,e,col="red")
   grid(col = "lightgray", lty = "dotted")
   dev.off()
}
smearPlot = function(DGEList,deTags, outSmear, outMain)
      {
      pdf(outSmear)
      plotSmear(DGEList,de.tags=deTags,main=outMain)
      grid(col="lightgray", lty="dotted")
      dev.off()
      }
boxPlot = function(rawrs,cleanrs,maint,myTitle,pdfname)
```

```
{           nc = ncol(rawrs)

        for (i in c(1:nc)) {rawrs[(rawrs[,i] < 0),i] = NA}

        fullnames = colnames(rawrs)

        newcolnames = substr(colnames(rawrs),1,20)

        colnames(rawrs) = newcolnames

        newcolnames = substr(colnames(cleanrs),1,20)

        colnames(cleanrs) = newcolnames

        defpar = par(no.readonly=T)

        print.noquote('raw contig counts by sample:')

        print.noquote(summary(rawrs))

        print.noquote('normalised contig counts by sample:')

        print.noquote(summary(cleanrs))

        pdf(pdfname)

        par(mfrow=c(1,2))

        boxplot(rawrs,varwidth=T,notch=T,ylab='log contig
count',col="maroon",las=3,cex.axis=0.35,main=paste('Raw:',maint))

        grid(col="lightgray",lty="dotted")

        boxplot(cleanrs,varwidth=T,notch=T,ylab='log contig
count',col="maroon",las=3,cex.axis=0.35,main=paste('After ',maint))

        grid(col="lightgray",lty="dotted")

        dev.off()

        pdfname = "sample_counts_histogram.pdf"

        nc = ncol(rawrs)

        print.noquote(paste('Using ncol rawrs=',nc))

        ncroot = round(sqrt(nc))

        if (ncroot*ncroot < nc) { ncroot = ncroot + 1 }
```

```
        m = c()
        for (i in c(1:nc)) {
              rhist = hist(rawrs[,i],breaks=100,plot=F)
              m = append(m,max(rhist$counts))
             }
        ymax = max(m)
        ncols = length(fullnames)
        if (ncols > 20)
        {
           scale = 7*ncols/20
           pdf(pdfname,width=scale,height=scale)
        } else {
           pdf(pdfname)
        }
        par(mfrow=c(ncroot,ncroot))
        for (i in c(1:nc)) {
                 hist(rawrs[,i], main=paste("Contig logcount",i), xlab='log raw count',
col="maroon",
                 breaks=100,sub=fullnames[i],cex=0.8,ylim=c(0,ymax))
             }
        dev.off()
        par(defpar)
}
cumPlot = function(rawrs,cleanrs,maint,myTitle)
{   # updated to use ecdf
        pdfname = "Filtering_rowsum_bar_charts.pdf"
```

```
        defpar = par(no.readonly=T)
        lrs = log(rawrs,10)
        lim = max(lrs)
        pdf(pdfname)
        par(mfrow=c(2,1))
        hist(lrs,breaks=100,main=paste('Before:',maint),xlab="# Reads (log)",
             ylab="Count",col="maroon",sub=myTitle, xlim=c(0,lim),las=1)
        grid(col="lightgray", lty="dotted")
        lrs = log(cleanrs,10)
        hist(lrs,breaks=100,main=paste('After:',maint),xlab="# Reads (log)",
             ylab="Count",col="maroon",sub=myTitle,xlim=c(0,lim),las=1)
        grid(col="lightgray", lty="dotted")
        dev.off()
        par(defpar)
}

cumPlot1 = function(rawrs,cleanrs,maint,myTitle)
{   # updated to use ecdf
        pdfname = paste(gsub(" ","", myTitle , fixed=TRUE),"RowsumCum.pdf",sep='_')
        pdf(pdfname)
        par(mfrow=c(2,1))
        lastx = max(rawrs)
        rawe = knots(ecdf(rawrs))
        cleane = knots(ecdf(cleanrs))
        cy = 1:length(cleane)/length(cleane)
        ry = 1:length(rawe)/length(rawe)
```

```
        plot(rawe,ry,type='l',main=paste('Before',maint),xlab="Log Contig Total Reads",
            ylab="Cumulative proportion",col="maroon",log='x',xlim=c(1,lastx),sub=myTitle)
        grid(col="blue")
        plot(cleane,cy,type='l',main=paste('After',maint),xlab="Log Contig Total Reads",
            ylab="Cumulative proportion",col="maroon",log='x',xlim=c(1,lastx),sub=myTitle)
        grid(col="blue")
        dev.off()
}

edgeIt = function
(Count_Matrix=c(),group=c(),out_edgeR=F,out_VOOM=F,out_DESeq2=F,fdrtype='fdr',prior
df=5,
        fdrthresh=0.05,outputdir='.', myTitle='Differential Counts',libSize=c(),useNDF=F,
        filterquantile=0.2, subjects=c(),mydesign=NULL,
        doDESeq2=T,doVoom=T,doCamera=T,doedgeR=T,org='hg19',
        histgmt="",
bigmt="/data/genomes/gsea/3.1/Abetterchoice_nocgp_c2_c3_c5_symbols_all.gmt",
        doCook=F,DESeq_fitType="parameteric")
{
  # Error handling
  if (length(unique(group))!=2){
    print("Number of conditions identified in experiment does not equal 2")
    q()
    }
  require(edgeR)
  options(width = 512)
  mt = paste(unlist(strsplit(myTitle,'_')),collapse=" ")
```

```
  allN = nrow(Count_Matrix)

  nscut = round(ncol(Count_Matrix)/2)

  colTotmillionreads = colSums(Count_Matrix)/1e6

  counts.dataframe = as.data.frame(c())

  rawrs = rowSums(Count_Matrix)

  nonzerod = Count_Matrix[(rawrs > 0),] # remove all zero count genes

  nzN = nrow(nonzerod)

  nzrs = rowSums(nonzerod)

  zN = allN - nzN

  print('# Quantiles for non-zero row counts:',quote=F)

  print(quantile(nzrs,probs=seq(0,1,0.1)),quote=F)

  if (useNDF == T)

  {

    gt1rpin3 =
rowSums(Count_Matrix/expandAsMatrix(colTotmillionreads,dim(Count_Matrix)) >= 1) >=
nscut

    lo = colSums(Count_Matrix[!gt1rpin3,])

    workCM = Count_Matrix[gt1rpin3,]

    cleanrs = rowSums(workCM)

    cleanN = length(cleanrs)

    meth = paste( "After removing",length(lo),"contigs with fewer than ",nscut," sample read
counts >= 1 per million, there are",sep="")

    print(paste("Read",allN,"contigs. Removed",zN,"contigs with no
reads.",meth,cleanN,"contigs"),quote=F)

    maint = paste('Filter >=1/million reads in >=',nscut,'samples')

  }   else {

    useme = (nzrs > quantile(nzrs,filterquantile))
```

```
    workCM = nonzerod[useme,]

    lo = colSums(nonzerod[!useme,])

    cleanrs = rowSums(workCM)

    cleanN = length(cleanrs)

    meth = paste("After filtering at count quantile =",filterquantile,", there are",sep="")

    print(paste('Read',allN,"contigs. Removed",zN,"with no
reads.",meth,cleanN,"contigs"),quote=F)

    maint = paste('Filter below',filterquantile,'quantile')

  }

  cumPlot(rawrs=rawrs,cleanrs=cleanrs,maint=maint,myTitle=myTitle)

  allgenes = rownames(workCM)

  reg = "^chr([0-9]+):([0-9]+)-([0-9]+)"

  genecards="<a href=\'http://www.genecards.org/index.php?path=/Search/keyword/"

  ucsc = paste("<a href=\'http://genome.ucsc.edu/cgi-bin/hgTracks?db=",org,sep=")

  testreg = str_match(allgenes,reg)

  if (sum(!is.na(testreg[,1]))/length(testreg[,1]) > 0.8) # is ucsc style string

  {

    print("@@ using ucsc substitution for urls")

    contigurls = paste0(ucsc,"&amp;position=chr",testreg[,2],":",testreg[,3],"-
",testreg[,4],"\'>",allgenes,"</a>")

  } else {

    print("@@ using genecards substitution for urls")

    contigurls = paste0(genecards,allgenes,"\'>",allgenes,"</a>")

  }

  print.noquote("# urls")

  print.noquote(head(contigurls))
```

```
  print(paste("# Total low count contigs per sample = ",paste(lo,collapse=',')),quote=F)

  cmrowsums = rowSums(workCM)

  TName=unique(group)[1]

  CName=unique(group)[2]

  if (is.null(mydesign)) {

    if (length(subjects) == 0)

    {

      mydesign = model.matrix(~group)

    }

    else {

      subjf = factor(subjects)

      mydesign = model.matrix(~subjf+group) # we block on subject so make group last to
simplify finding it

    }

  }

  print.noquote(paste('Using samples:',paste(colnames(workCM),collapse=',')))

  print.noquote('Using design matrix:')

  print.noquote(mydesign)

  if (doedgeR) {

  sink('edgeR.log')

  DGEList = DGEList(counts=workCM, group = group)

  DGEList = calcNormFactors(DGEList)


  DGEList = estimateGLMCommonDisp(DGEList,mydesign)

  comdisp = DGEList$common.dispersion

  DGEList = estimateGLMTrendedDisp(DGEList,mydesign)
```

```
  if (edgeR_priordf > 0) {
    print.noquote(paste("prior.df =",edgeR_priordf))
    DGEList = estimateGLMTagwiseDisp(DGEList,mydesign,prior.df = edgeR_priordf)
  } else {
    DGEList = estimateGLMTagwiseDisp(DGEList,mydesign)
  }
  DGLM = glmFit(DGEList,design=mydesign)
  DE = glmLRT(DGLM,coef=ncol(DGLM$design)) # always last one - subject is first if
needed
  efflib = DGEList$samples$lib.size*DGEList$samples$norm.factors
  normData = (1e+06*DGEList$counts/efflib)
  uoutput = cbind(
    Name=as.character(rownames(DGEList$counts)),
    DE$table,
    adj.p.value=p.adjust(DE$table$PValue, method=fdrtype),
    Dispersion=DGEList$tagwise.dispersion,totreads=cmrowsums,normData,
    DGEList$counts
  )
  soutput = uoutput[order(DE$table$PValue),] # sorted into p value order - for quick toptable
  goodness = gof(DGLM, pcutoff=fdrthresh)
  if (sum(goodness$outlier) > 0) {
    print.noquote('GLM outliers:')
    print(paste(rownames(DGLM)[(goodness$outlier)],collapse=','),quote=F)
    } else {
      print('No GLM fit outlier genes found\n')
```

```
  }

 z = limma::zscoreGamma(goodness$gof.statistic, shape=goodness$df/2, scale=2)

 pdf("edgeR_GoodnessofFit.pdf")

 qq = qqnorm(z, panel.first=grid(), main="tagwise dispersion")

 abline(0,1,lwd=3)

 points(qq$x[goodness$outlier],qq$y[goodness$outlier], pch=16, col="maroon")

 dev.off()

 estpriorn = getPriorN(DGEList)

 print(paste("Common Dispersion =",comdisp,"CV = ",sqrt(comdisp),"getPriorN =
",estpriorn),quote=F)

 efflib = DGEList$samples$lib.size*DGEList$samples$norm.factors

 normData = (1e+06*DGEList$counts/efflib)

 uniqueg = unique(group)

 sample_colors =  match(group,levels(group))

 sampleTypes = levels(factor(group))

 print.noquote(sampleTypes)

 pdf("edgeR_MDSplot.pdf")

 plotMDS.DGEList(DGEList,main=paste("edgeR MDS
for",myTitle),cex=0.5,col=sample_colors,pch=sample_colors)

 legend(x="topleft", legend = sampleTypes,col=c(1:length(sampleTypes)), pch=19)

 grid(col="blue")

 dev.off()

 colnames(normData) = paste( colnames(normData),'N',sep="_")

 print(paste('Raw sample read totals',paste(colSums(nonzerod,na.rm=T),collapse=',')))

 nzd = data.frame(log(nonzerod + 1e-2,10))

 try( boxPlot(rawrs=nzd,cleanrs=log(normData,10),maint='TMM
Normalisation',myTitle=myTitle,pdfname="edgeR_raw_norm_counts_box.pdf") )
```

```
write.table(soutput,file=out_edgeR, quote=FALSE, sep="\t",row.names=F)

tt = cbind(

  Name=as.character(rownames(DGEList$counts)),

  DE$table,

  adj.p.value=p.adjust(DE$table$PValue, method=fdrtype),

  Dispersion=DGEList$tagwise.dispersion,totreads=cmrowsums

)

print.noquote("# edgeR Top tags\n")

tt = cbind(tt,URL=contigurls) # add to end so table isn't laid out strangely

tt = tt[order(DE$table$PValue),]

print.noquote(tt[1:50,])

deTags = rownames(uoutput[uoutput$adj.p.value < fdrthresh,])

nsig = length(deTags)

print(paste('#',nsig,'tags significant at adj p=',fdrthresh),quote=F)

deColours = ifelse(deTags,'red','black')

pdf("edgeR_BCV_vs_abundance.pdf")

plotBCV(DGEList, cex=0.3, main="Biological CV vs abundance")

dev.off()

dg = DGEList[order(DE$table$PValue),]

#normData = (1e+06 * dg$counts/expandAsMatrix(dg$samples$lib.size, dim(dg)))

efflib = dg$samples$lib.size*dg$samples$norm.factors

normData = (1e+06*dg$counts/efflib)

outpdfname="edgeR_top_100_heatmap.pdf"

hmap2(normData,nsamp=100,TName=TName,group=group,outpdfname=outpdfname,myTitl
e=paste('edgeR Heatmap',myTitle))
```

```
  outSmear = "edgeR_smearplot.pdf"

  outMain = paste("Smear Plot for ",TName,' Vs ',CName,' (FDR@',fdrthresh,' N =
',nsig,')',sep='')

  smearPlot(DGEList=DGEList,deTags=deTags, outSmear=outSmear, outMain = outMain)

  qqPlot(descr=paste(myTitle,'edgeR adj p QQ
plot'),pvector=tt$adj.p.value,outpdf='edgeR_qqplot.pdf')

  norm.factor = DGEList$samples$norm.factors

  topresults.edgeR = soutput[which(soutput$adj.p.value < fdrthresh), ]

  edgeRcountsindex = which(allgenes %in% rownames(topresults.edgeR))

  edgeRcounts = rep(0, length(allgenes))

  edgeRcounts[edgeRcountsindex] = 1  # Create venn diagram of hits

  sink()

  }

  if (doDESeq2 == T)

  {

   sink("DESeq2.log")

   # DESeq2

   require('DESeq2')

   library('RColorBrewer')

   if (length(subjects) == 0)

       {

       pdata =
data.frame(Name=colnames(workCM),Rx=group,row.names=colnames(workCM))

       deSEQds = DESeqDataSetFromMatrix(countData = workCM,  colData = pdata, design
= formula(~ Rx))

       } else {

       pdata =
```

```
data.frame(Name=colnames(workCM),Rx=group,subjects=subjects,row.names=colnames(wo
rkCM))

        deSEQds = DESeqDataSetFromMatrix(countData = workCM,  colData = pdata, design
= formula(~ subjects + Rx))

        }

    #DESeq2 = DESeq(deSEQds,fitType='local',pAdjustMethod=fdrtype)

    #rDESeq = results(DESeq2)

    #newCountDataSet(workCM, group)

    deSeqDatsizefac = estimateSizeFactors(deSEQds)

    deSeqDatdisp = estimateDispersions(deSeqDatsizefac,fitType=DESeq_fitType)

    resDESeq = nbinomWaldTest(deSeqDatdisp, pAdjustMethod=fdrtype)

    rDESeq = as.data.frame(results(resDESeq))

    rDESeq =
cbind(Contig=rownames(workCM),rDESeq,NReads=cmrowsums,URL=contigurls)

    srDESeq = rDESeq[order(rDESeq$pvalue),]

    qqPlot(descr=paste(myTitle,'DESeq2 adj p qq
plot'),pvector=rDESeq$padj,outpdf='DESeq2_qqplot.pdf')

    cat("# DESeq top 50\n")

    print.noquote(srDESeq[1:50,])

    write.table(srDESeq,file=out_DESeq2, quote=FALSE, sep="\t",row.names=F)

    topresults.DESeq = rDESeq[which(rDESeq$padj < fdrthresh), ]

    DESeqcountsindex = which(allgenes %in% rownames(topresults.DESeq))

    DESeqcounts = rep(0, length(allgenes))

    DESeqcounts[DESeqcountsindex] = 1

    pdf("DESeq2_dispersion_estimates.pdf")

    plotDispEsts(resDESeq)

    dev.off()
```

```
    ysmall = abs(min(rDESeq$log2FoldChange))
    ybig = abs(max(rDESeq$log2FoldChange))
    ylimit = min(4,ysmall,ybig)
    pdf("DESeq2_MA_plot.pdf")
    plotMA(resDESeq,main=paste(myTitle,"DESeq2 MA plot"),ylim=c(-ylimit,ylimit))
    dev.off()
    rlogres = rlogTransformation(resDESeq)
    sampledists = dist( t( assay(rlogres) ) )
    sdmat = as.matrix(sampledists)
    pdf("DESeq2_sample_distance_plot.pdf")
    heatmap.2(sdmat,trace="none",main=paste(myTitle,"DESeq2 sample distances"),
        col = colorRampPalette( rev(brewer.pal(9, "RdBu")) )(255))
    dev.off()
    sink()
    result = try( (ppca = plotPCA( varianceStabilizingTransformation(deSeqDatdisp,blind=T),
intgroup=c("Rx","Name")) ) )
    if ("try-error" %in% class(result)) {
        print.noquote('DESeq2 plotPCA failed.')
        } else {
        pdf("DESeq2_PCA_plot.pdf")
        print(ppca)
        dev.off()
       }
  }
  if (doVoom == T) {
     sink('VOOM.log')
```

```
if (doedgeR == F) {
  DGEList = DGEList(counts=workCM, group = group)
  DGEList = calcNormFactors(DGEList)
  DGEList = estimateGLMCommonDisp(DGEList,mydesign)
  DGEList = estimateGLMTrendedDisp(DGEList,mydesign)
  DGEList = estimateGLMTagwiseDisp(DGEList,mydesign)
  DGEList = estimateGLMTagwiseDisp(DGEList,mydesign)
  norm.factor = DGEList$samples$norm.factors
  }
pdf("VOOM_mean_variance_plot.pdf")
dat.voomed = voom(DGEList, mydesign, plot = TRUE, lib.size = colSums(workCM) *
norm.factor)
dev.off()
# Use limma to fit data
fit = lmFit(dat.voomed, mydesign)
fit = eBayes(fit)
rvoom = topTable(fit, coef = length(colnames(mydesign)), adj = fdrtype, n = Inf,
sort="none")
qqPlot(descr=paste(myTitle,'VOOM-limma adj p QQ
plot'),pvector=rvoom$adj.P.Val,outpdf='VOOM_qqplot.pdf')
rownames(rvoom) = rownames(workCM)
rvoom = cbind(rvoom,NReads=cmrowsums,URL=contigurls)
srvoom = rvoom[order(rvoom$P.Value),]
cat("# VOOM top 50\n")
print(srvoom[1:50,])
write.table(srvoom,file=out_VOOM, quote=FALSE, sep="\t",row.names=F)
```

```
    # Use an FDR cutoff to find interesting samples for edgeR, DESeq and voom/limma
    topresults.voom = rvoom[which(rvoom$adj.P.Val < fdrthresh), ]
    voomcountsindex = which(allgenes %in% topresults.voom$ID)
    voomcounts = rep(0, length(allgenes))
    voomcounts[voomcountsindex] = 1
    sink()
  }
 if ((doDESeq2==T) || (doVoom==T) || (doedgeR==T)) {
   if ((doVoom==T) && (doDESeq2==T) && (doedgeR==T)) {
      vennmain = paste(mt,'Voom,edgeR and DESeq2 overlap at FDR=',fdrthresh)
      counts.dataframe = data.frame(edgeR = edgeRcounts, DESeq2 = DESeqcounts,
                                    VOOM_limma = voomcounts, row.names = allgenes)
     } else if ((doDESeq2==T) && (doedgeR==T))  {
       vennmain = paste(mt,'DESeq2 and edgeR overlap at FDR=',fdrthresh)
       counts.dataframe = data.frame(edgeR = edgeRcounts, DESeq2 = DESeqcounts,
row.names = allgenes)
     } else if ((doVoom==T) && (doedgeR==T)) {
      vennmain = paste(mt,'Voom and edgeR overlap at FDR=',fdrthresh)
      counts.dataframe = data.frame(edgeR = edgeRcounts, VOOM_limma = voomcounts,
row.names = allgenes)
     }

  if (nrow(counts.dataframe > 1)) {
    counts.venn = vennCounts(counts.dataframe)
    vennf = "Venn_significant_genes_overlap.pdf"
    pdf(vennf)
    vennDiagram(counts.venn,main=vennmain,col="maroon")
```

```
      dev.off()
    }
  }
}

builtin_gmt = ""
history_gmt = ""
history_gmt_name = ""
out_edgeR = F
out_DESeq2 = F
out_VOOM = "None"
doDESeq2 = F # make these T or F
doVoom = F
doCamera = F
doedgeR = T
edgeR_priordf = 0
  out_edgeR =
"/data/extended/galaxy_data_store/job_working_directory/029/29194/galaxy_dataset_37281.
dat"
  edgeR_priordf = 20

if (sum(c(doedgeR,doVoom,doDESeq2)) == 0)
{
write("No methods chosen - nothing to do! Please try again after choosing one or more
methods", stderr())
quit(save="no",status=2)
}
```

```
Out_Dir =
"/data/extended/galaxy_data_store/job_working_directory/029/29194/dataset_37282_files"

Input =  "/data/extended/galaxy_data_store/files/037/dataset_37280.dat"

TreatmentName = "R"

TreatmentCols = "10,11,12,13,14,15,16,17"

ControlName = "WT"

ControlCols= "2,3,4,5,6,7,8,9"

org = "mm9"

if (org == "") { org = "hg19"}

fdrtype = "fdr"

fdrthresh = 0.05

useNDF = F

fQ = 0.3 # non-differential centile cutoff

myTitle = "ArrhusmousedataDGE"

sids = strsplit("",',')

subjects = unlist(sids)

nsubj = length(subjects)

TCols = as.numeric(strsplit(TreatmentCols,",")[[1]])-1

CCols = as.numeric(strsplit(ControlCols,",")[[1]])-1

cat('Got TCols=')

cat(TCols)

cat('; CCols=')

cat(CCols)

cat('\n')

useCols = c(TCols,CCols)

if (file.exists(Out_Dir) == F) dir.create(Out_Dir)
```

```
Count_Matrix = read.table(Input,header=T,row.names=1,sep='\t') #Load tab file assume header

snames = colnames(Count_Matrix)

nsamples = length(snames)

if (nsubj >  0 & nsubj != nsamples) {

options("show.error.messages"=T)

mess = paste('Fatal error: Supplied subject id list',paste(subjects,collapse=','),

   'has length',nsubj,'but there are',nsamples,'samples',paste(snames,collapse=','))

write(mess, stderr())

quit(save="no",status=4)

}

if (length(subjects) != 0) {subjects = subjects[useCols]}

Count_Matrix = Count_Matrix[,useCols]

rn = rownames(Count_Matrix)

islib = rn %in% c('librarySize','NotInBedRegions')

LibSizes = Count_Matrix[subset(rn,islib),][1] # take first

Count_Matrix = Count_Matrix[subset(rn,! islib),]

group = c(rep(TreatmentName,length(TCols)), rep(ControlName,length(CCols)) )
#Build a group descriptor

group = factor(group, levels=c(ControlName,TreatmentName))

colnames(Count_Matrix) = paste(group,colnames(Count_Matrix),sep="_")
#Relable columns

results = edgeIt(Count_Matrix=Count_Matrix,group=group, out_edgeR=out_edgeR,
out_VOOM=out_VOOM, out_DESeq2=out_DESeq2,


fdrtype='BH',mydesign=NULL,priordf=edgeR_priordf,fdrthresh=fdrthresh,outputdir='.',

             myTitle=myTitle,useNDF=F,libSize=c(),filterquantile=fQ,subjects=subjects,
```

```
doDESeq2=doDESeq2,doVoom=doVoom,doCamera=doCamera,doedgeR=doedgeR,org=org,
        histgmt=history_gmt,bigmt=builtin_gmt,DESeq_fitType=DESeq_fitType)
sessionInfo()
```

5 Cuffdiff2 Analysis

Reads that passed quality control (more than 90% bases had less than 1% sequencing error; no ambiguous bases) were aligned to the mouse genome, Mus_musculus.GRCm.38.72, with corresponding gene model annotation file, Mus_musculus.GRCm38.72.gtf, by TopHat 2.0.6. Tophat2 was run using a Galaxy web tool available at https://toolshed.g2.bx.psu.edu/view/devteam/tophat2/ffa30bedbee3 (version 0:ffa30bedbee3) with the following options,

1. **Library**: single end
2. **Use a built in reference genome or own from your history**: Use a genome from history
3. **Select the reference genome**: Mus_musculus.GRCm.38.72 FASTA file
4. **TopHat settings to use**: Full parameter list
5. **Max realign edit distance**: 0
6. **Max edit distance**: 2
7. **Library Type**: FR unstranded
8. **Final read mismatches**: 2
9. **Use bowtie -n mode**: No
10. **Anchor length**: 8
11. **Maximum number of mismatches that can appear in the anchor region of spliced alignment**: 0
12. **The minimum intron length**: 70
13. **The maximum intron length**: 500000
14. **Allow indel search**: Yes
15. **Max insertion length**: 3
16. **Max deletion length:** 3
17. **Maximum number of alignments to be allowed**: 20
18. **Number of mismatches allowed in each segment alignment for reads mapped independently**: 2
19. **Minimum length of read segments**: 25
20. **Use Own Junctions**: Yes
21. **Use Gene Annotation Model**: Yes
22. **Gene Model Annotations**: Mus_musculus.GRCm38.72.gtf file

23. **Use Coverage Search**: Yes
24. **Minimum intron length that may be found during coverage search**: 50
25. **Maximum intron length that may be found during coverage search**: 20000
26. **Use Microexon Search**: No

Tophat2 aligned reads were assembled into transcripts using Cufflinks 2.1.1 that was run using a Galaxy web tool available at https://toolshed.g2.bx.psu.edu/view/devteam/cufflinks/7498f5a8fe06 (version 3:7498f5a8fe06) with the following options,

1. **Max Intron Length:** 300000
2. **Min Isoform Fraction**: 0.1
3. **Pre MRNA Fraction**: 0.15
4. **Perform quartile normalization**: Yes
5. **Use Reference Annotation**: Use Reference Annotation
6. **Reference Annotation**: Mus_musculus.GRCm38.72.gtf file
7. **Perform Bias Correction**: Yes
8. **Reference sequence data**: History
9. **Using reference file**: Mus_musculus.GRCm.38.72 FASTA file
10. **Use multi-read correct**: Yes
11. **Use effective length correction**: Yes

Aligned transcripts files were combined by Cuffmerge 2.1.1 that was run using a Galaxy web tool available at https://toolshed.g2.bx.psu.edu/view/devteam/cuffmerge/aa209d311ece (version 3:aa209d311ece) with the following options,

1. **Use Reference Annotation**: Yes
2. **Reference Annotation**: Mus_musculus.GRCm38.72.gtf file
3. **Use Sequence Data**: Yes
4. **Choose the source for the reference list**: History
5. **Using reference file**: Mus_musculus.GRCm.38.72 FASTA file

Then, we identified differentially expressed genes by Cuffdiff2 that was run using a Galaxy web tool available at https://toolshed.g2.bx.psu.edu/view/devteam/cuffdiff/2d6a90609943 (version 2:2d6a90609943) with the following options,

1. **Library normalization method**: Geometric
2. **Dispersion estimation method**: per-condition
3. **False Discovery Rate**: 0.05
4. **Min Alignment Count**: 10
5. **Use multi-read correct**: Yes
6. **Perform Bias Correction**: Yes
7. **Reference sequence data**: History
8. **Using reference file**: Mus_musculus.GRCm.38.72 FASTA file
9. **Include Read Group Datasets**: Yes
