# Supplementary material for: Experimental validation of methods for differential gene expression analysis and sample pooling in RNA-seq
Source: BMC Genomics. 2015 Jul 25;16(1):548. doi: 10.1186/s12864-015-1767-y (PMC4515013; doi:10.1186/s12864-015-1767-y)
Supplement: Additional file 12: — A table presenting overview of RNA extraction, cDNA synthesis, specific target amplification and high-throughput qPCR procedures. [file 12864_2015_1767_MOESM12_ESM.docx]

**Table E8**: Overview of RNA extraction, cDNA synthesis, specific target amplification and high-throughput qPCR procedures

| **Procedure** | **Methods** | **Quality control** |
| --- | --- | --- |
| **RNA extraction** | Maxwell-16 system and LEV simplyRNA Tissue Kit (Promega, Madison, USA), using 200µl of homogenization solution, 200µl of lysis buffer, 5µl of DNase I, 50µl of nuclease free water, and simply RNA protocol (Promega, Madison, USA) | 260/280 ratio, measured by NanoDrop 1000 version 3.7.1 (Thermo Fisher Scientific, Waltham, MA, USA) above 1.8 |
| **cDNA synthesis** | iScript select cDNA Synthesis Kit (Bio-Rad, Hercules, USA), using 180ng of RNA, and 1:1 mixture of oligo(dT) and random primers. cDNA synthesis incubation program: 25°C for 5 minutes (min), 42°C for 90 min, and 85°C for 5 min | Employed corresponding negative controls without iScript reverse transcriptase |
| **Specific target amplification (STA)** | 48 forward and 48 reverse primers (100 µM/l) were mixed and diluted to 500nM/l/primer, using DNA suspension buffer (Teknova, Hollister, CA, USA). STA (95°C for 2 min, and 10-20 cycles of 95°C for 15 sec and 60°C for 4 min) with 1µl of PreAmp master mix (Fluidigm, San Francisco, USA), 0.5µl of diluted primer mix, 1.25µl of cDNA, and 2.25µl of nuclease free water. | Employed corresponding no-template negative controls without cDNA |
| **Exonuclease I treatment** | Unincorporated primers were removed by Exonuclease I at 20 Units/μL (New England BioLabs, Ipswich, MA, USA). Treatment incubation program: 37°C for 30 min, and 80°C for 15 min |  |
| **Procedure** | **Methods** | **Quality control** |
| **Preparing serially diluted standards** | Pooled cDNA samples (16 samples from both groups; 2µl/sample) underwent STA and Exonuclease I treatment. Seven two-fold serial dilutions (1:2 to 1:128) were made by adding TE buffer (Teknova, Hollister, CA, USA) |  |
| **High-throughput quantitative polymerase chain reaction (qPCR)** | BioMark HD (Fluidigm, San Francisco, USA), using 48.48 dynamic arrays (Fluidigm, San Francisco, USA), SsoFast EvaGreen Low ROX kit (Bio-Rad, Hercules, USA), 20X DNA binding dye sample loading reagent, and 2X Assay loading reagent (Fluidigm, San Francisco, USA). GE Fast 48x48 PCR+Melt v2 thermal cycling protocol: 95°C for 1 min, and 35 cycles of 96°C for 5 sec and 60°C for 20 sec. | Melting curve analysis to assess the specificity of the primers (60-95°C; 1°C/ 3 sec). Serially diluted standards were loaded in duplicates. No-reverse transcriptase and no-template negative controls were included in all arrays. |
